# Crystal Structures of XeF_2_·2PtF_4_ and XeF_2_·2PdF_4_ Determined by 3D Electron Diffraction and Structural Models of XePtF_6_

**DOI:** 10.1021/acs.inorgchem.5c01740

**Published:** 2025-07-14

**Authors:** Klemen Motaln, Kshitij Gurung, Mirela Dragomir, Dominik Kurzydłowski, Lukáš Palatinus, Matic Lozinšek

**Affiliations:** † Jožef Stefan Institute, Jamova cesta 39, 1000 Ljubljana, Slovenia; ‡ Jožef Stefan International Postgraduate School, Jamova cesta 39, 1000 Ljubljana, Slovenia; § Department of Structure Analysis, Institute of Physics of the Czech Academy of Sciences, Na Slovance 1999/2, Prague 8 18221, Czech Republic; ∥ Faculty of Mathematics and Natural Sciences, Cardinal Stefan Wyszyński University in Warsaw, 01-938 Warsaw, Poland

## Abstract

Although the demonstration of noble-gas reactivity represents one of the most significant breakthroughs of 20th-century inorganic chemistry, the first noble-gas compound, XePtF_6_ (XeF_2_·PtF_4_), lacks comprehensive structural characterization, and its structure remains to be elucidated. In this study, the XeF_2_–PtF_4_ and XeF_2_–PdF_4_ systems were reexplored, resulting in the crystal structure determination of XeF_2_·2PtF_4_ and XeF_2_·2PdF_4_ by 3D electron diffraction, marking the first successful structural characterization of compounds from these systems. Both compounds are isostructural with the previously characterized XeF_2_·2MnF_4_, featuring corrugated zigzag double-chain motifs formed by interconnected octahedral fluoridometallate­(IV) units. Periodic density functional theory calculations were employed to evaluate the structural models of XeF_2_·PtF_4_, which were derived from experimentally determined crystal structures of XeF_2_–MF_4_ (M = Cr, Mn) analogues. The results reveal a preference for *cis*-bridging between adjacent platinum­(IV) centers and show that a tetrameric ring structure and *cis*-chain polymorph, modeled after the crystal structure of XeF_2_·MnF_4_ and XeF_2_-deficient 3XeF_2_·2MnF_4_, respectively, emerge as energetically favored. The results of this study thus provide a direct structural link between platinum, palladium, and manganese analogues in the XeF_2_–MF_4_ systems and highlight the tetrameric ring structure and *cis*-chain as likely structural models of XeF_2_·PtF_4_.

## Graphical abstract

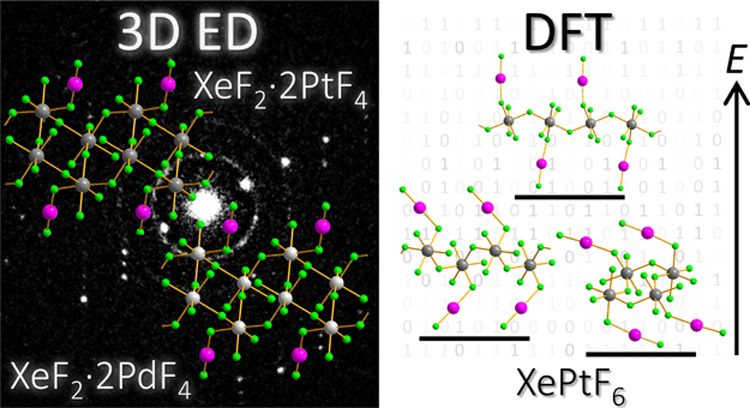

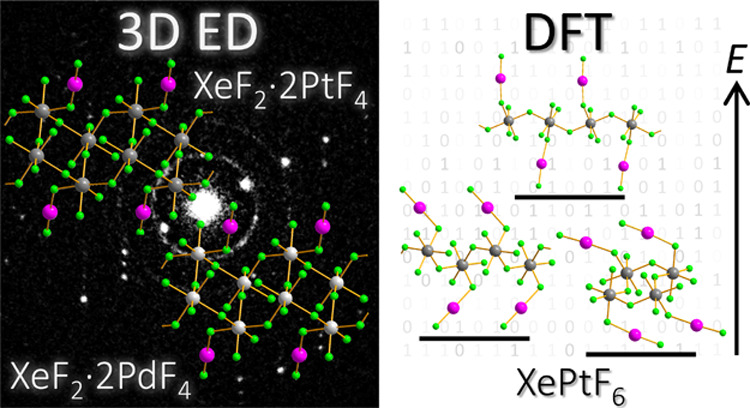

## 1 Introduction

The dogma of the chemical inertness of noble gases was dispelled in 1962 by a landmark experiment carried out by Neil Bartlett, where he oxidized and fluorinated gaseous xenon by PtF_6_.^1^ This daring experiment was inspired^2^ by his earlier observation that PtF_6_ oxidizes O_2_ gas,^3^ identifying PtF_6_ as an extremely powerful oxidizing agent. The analysis of the yellow solid deposited in the reaction of red PtF_6_ vapors with colorless Xe gas revealed that the stoichiometry of the product was XePtF_6_.^1,4^ A subsequent reinvestigation of this reaction indicated that the compound is likely diamagnetic, thus corresponding to oxidation states of +4 for platinum and +2 for xenon, and can therefore be represented as a Lewis acid–base adduct, XeF_2_·PtF_4_.^4^ It has been conjectured that the structure of this compound may consist of a F-bridged fluoridoplatinate backbone, which could adopt the form of either a chain polymer or a discrete ring, coordinated by XeF_2_ molecules.^4^ However, due to its inherently amorphous nature, no definitive structural information on this fascinating compound could ever be obtained, despite its great historical significance.^4,5^ To complicate matters further, compounds of pentavalent platinum are also formed when Xe gas is oxidized by PtF_6_, especially when an excess of the latter is used, as evidenced by the observation of the powder X-ray diffraction pattern^6^ of [XeF]­[PtF_6_] during various preparations of XePtF_6_.^4,5,7^ Both PtF_5_ and PtF_4_ can form adducts with XeF_2_, and in addition to the aforementioned XeF_2_·PtF_4_ and [XeF]­[PtF_6_]; [Xe_2_F_3_]­[PtF_6_], [XeF]­[Pt_2_F_11_],^4,6–8^ and XeF_2_·2PtF_4_ ^4,5,7^ have also been synthesized and vibrationally characterized. Unfortunately, no crystal structures of any of the aforementioned compounds have been reported to date.

The structures of platinum­(V) compounds are expected to be analogous to the well-characterized pnictogen­(V) analogues, namely ionic [Xe_2_F_3_]^+^[PnF_6_]^−^ (Pn = As, Sb),^8,9^ and molecular species [XeF]^+^[PnF_6_]^−^ and [XeF]^+^[Pn_2_F_11_]^−^ (Pn = As, Sb, Bi),^10^ which are better described as tight ion pairs with the anion F-coordinated to the strongly Lewis acidic XeF^+^ cation. On the other hand, the investigations of XeF_2_–MF_4_ adducts as chemical analogues to XeF_2_·PtF_4_, where M = Ti,^11–13^ Cr,^14–16^ Mn,^17–19^ Rh,^5^ Pd,^7^ and Sn,^20^ have established the existence of products with a wide range of stoichiometries.^21^ Despite the fact that only a handful of crystal structures have been reported, these adducts display a remarkable structural diversity (Figure 1). A *trans*-chain of [CrF_6_] octahedra, each coordinated by a XeF_2_ ligand, is observed in XeF_2_·CrF_4_, whereas in the XeF_2_·2CrF_4_ adduct, tetrameric rings are interconnected into a layered structure. The metal-rich adducts from the XeF_2_–TiF_4_ system display a high degree of XeF_2_ ionization^22^ and are therefore better described as salts of Xe_2_F_3_^+^ and XeF^+^. Octameric cube-like units are connected into a layer in [Xe_2_F_3_]­[Ti_8_F_33_] (XeF_2_·4TiF_4_), whereas the structure of [XeF]_2_[Ti_9_F_38_] (2XeF_2_·9TiF_4_) features nonameric trigonal prismatic units connected by fluoride bridges into chains.^13^ In a recent reinvestigation of the XeF_2_–MnF_4_ system, the crystal structures of 3XeF_2_·2MnF_4_, XeF_2_·MnF_4_, and XeF_2_·2MnF_4_ were determined by both single-crystal X-ray diffraction (SCXRD) and 3D electron diffraction (3D ED).^19^ The successful application of 3D ED to the study of these reactive compounds is of particular importance, as the previous structural investigations of the XeF_2_–MF_4_ family of compounds were often impeded by the inability to prepare single crystals of sufficient size and quality for SCXRD. The crystal structures of 3XeF_2_·2MnF_4_ and XeF_2_·2MnF_4_ were found to consist of infinite polymeric chains and double chains, respectively, whereas discrete tetrameric rings were observed in the structure of XeF_2_·MnF_4_. Among the structurally characterized XeF_2_–MF_4_ compounds, only XeF_2_·CrF_4_ and XeF_2_·MnF_4_ represent the XeMF_6_ stoichiometry observed in the first noble-gas compound XePtF_6_, which renders them potential structural models for the structure of XeF_2_·PtF_4_, although various other structural types are also possible.

**Figure 1:**
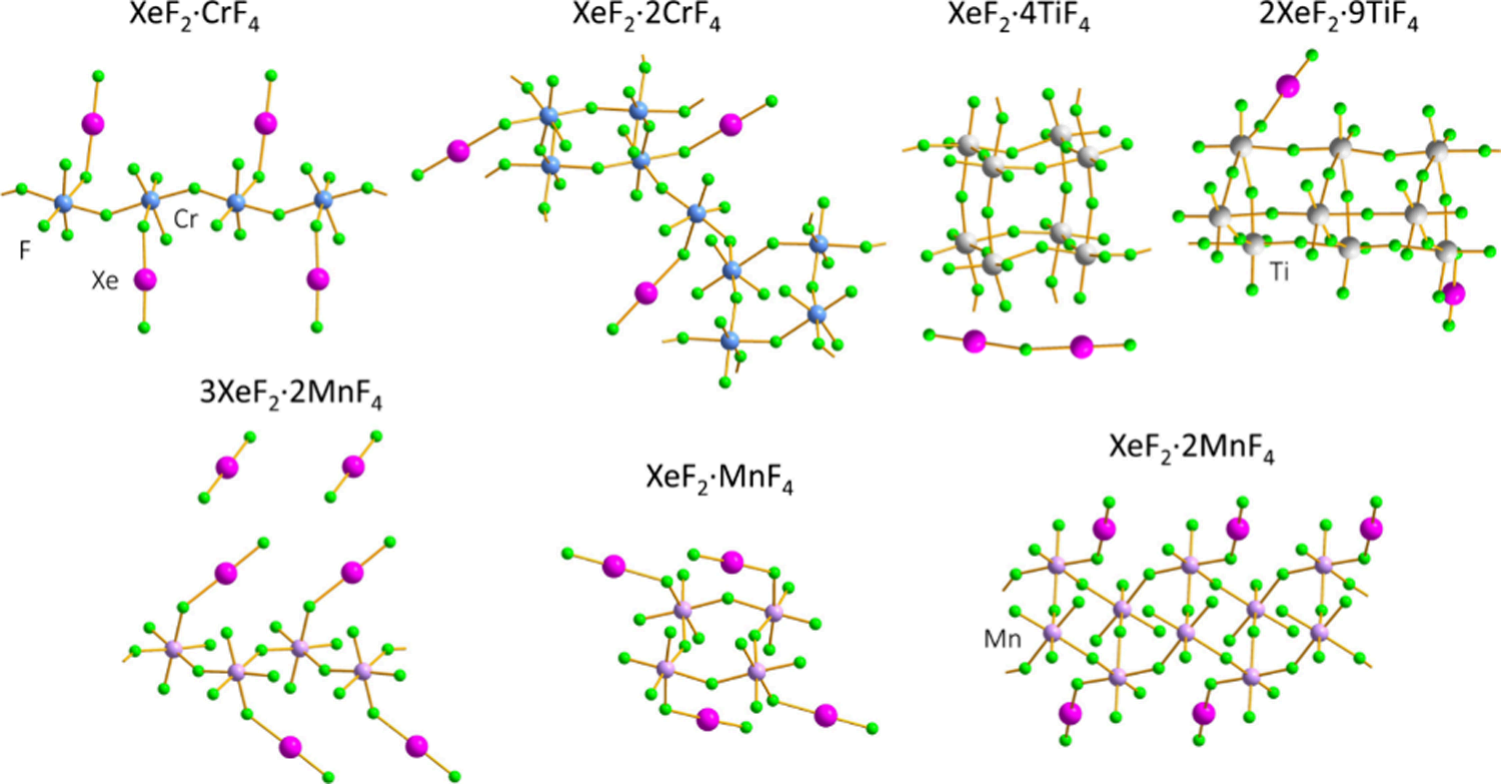
Rich structural diversity exhibited by crystallographically characterized XeF_2_–MF_4_ compounds.^13,15,16,19^

In this work, the XeF_2_–PdF_4_ and XeF_2_–PtF_4_ systems were reinvestigated, leading to the first successful structural characterization of compounds from these systems, with the crystal structures of XeF_2_·2PdF_4_ and XeF_2_·2PtF_4_ elucidated by 3D ED. The two compounds were revealed to be isostructural with XeF_2_·2MnF_4_, thus establishing a direct structural link between the well-characterized XeF_2_–MnF_4_ system and its platinum and palladium analogues. Moreover, structural models for the crystal structure of the first discovered noble-gas compound XePtF_6_ (XeF_2_·PtF_4_) are proposed based on DFT calculations augmented by evolutionary structure searches.

## 2 Results and Discussion

### 2.1 Syntheses

The reaction of XeF_2_ with platinum powder in aHF media (Figure 2) was previously reported as the first step of a convenient method for synthesizing PtF_4_. The initially formed orange solid could be pyrolyzed at 300 °C to yield pure PtF_4_, although the nature of the intermediate orange product was not investigated.^23^ During the course of this study, the orange-red solid that forms after the removal of the solvent was isolated at temperatures between −40 and −60 °C. The material obtained was found to be a mixture of XeF_2_ and an amorphous orange-red solid, which exhibited only broad bands in its Raman spectrum (Figure 3). Nevertheless, the positions of the strongest bands, located at 655 and 588 cm^–1^, were found to agree reasonably well with the reported Raman spectrum of the XePtF_6_ prepared by the reaction of excess XeF_2_ with PtF_4_ in anhydrous HF, which exhibited three lines: 657 cm^–1^ (very strong), 591 cm^–1^ (strong), and 480 cm^–1^ (weak).^4^ When the orange-red product investigated in this study was heated to a temperature of 100 °C under dynamic vacuum, the release of XeF_2_ was observed, which was accumulated in an FEP trap held at −196 °C. The orange pyrolysis product was again found to be amorphous (Figure S1) and exhibited a Raman spectrum that was highly similar to that of the initial orange-red phase, with the exception of the band at 504 cm^–1^, which was no longer present (Figure 3). Furthermore, a slight change in the position of some of the bands is evident. As it was observed by Bartlett et al. that reactions of excess XeF_2_ with PtF_4_ in aHF produced orange-colored adducts, with the molar ratio of XeF_2_:PtF_4_ as high as 1.6:1,^4^ it can be assumed, that the initially formed orange-red solid is likely a XeF_2_ “rich” phase with a stoichiometry of *n*XeF_2_·PtF_4_ where 1 < *n* < 1.6 (eq 1), which loses the weakly bound XeF_2_ at elevated temperatures under dynamic vacuum, yielding a phase with composition ∼XeF_2_·PtF_4_ (eq 2).

$$\mathrm{Pt}+(n+2){\mathrm{XeF}}_{2}\overset{\mathrm{aHF}}{\to }n{\mathrm{XeF}}_{2}\cdot {\mathrm{PtF}}_{4}+2\mathrm{Xe}\;(1<n<1.6)$$ 1

nXeF2·PtF4→dynamicvacuum100C°XeF2·PtF4+(n−1)XeF2(1<n<1.6) 2

**Figure 2:**
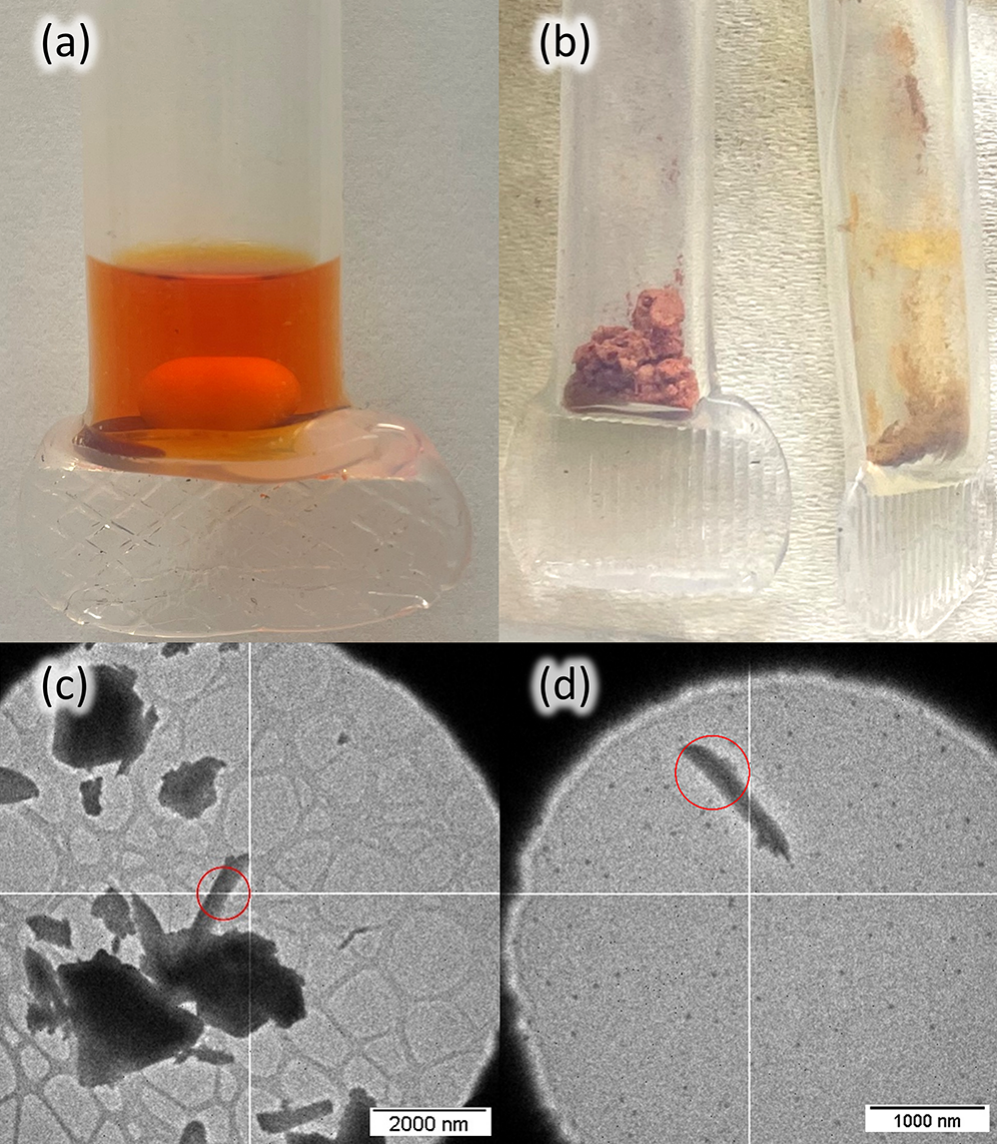
(a) Bright orange-colored solution formed when XeF_2_ oxidizes Pt powder in aHF (19 mm o.d. FEP vessel). (b) Isolated XeF_2_·2PtF_4_ (left) and XeF_2_·2PdF_4_ (right) powder samples stored in FEP containers (8 mm o.d.), showing their vivid peach and orange-brown colors, respectively. (c,d) Crystallites of XeF_2_·2PtF_4_ and XeF_2_·2PdF_4_, respectively, used for structural determination by 3D ED, as observed in the TEM.

**Figure 3:**
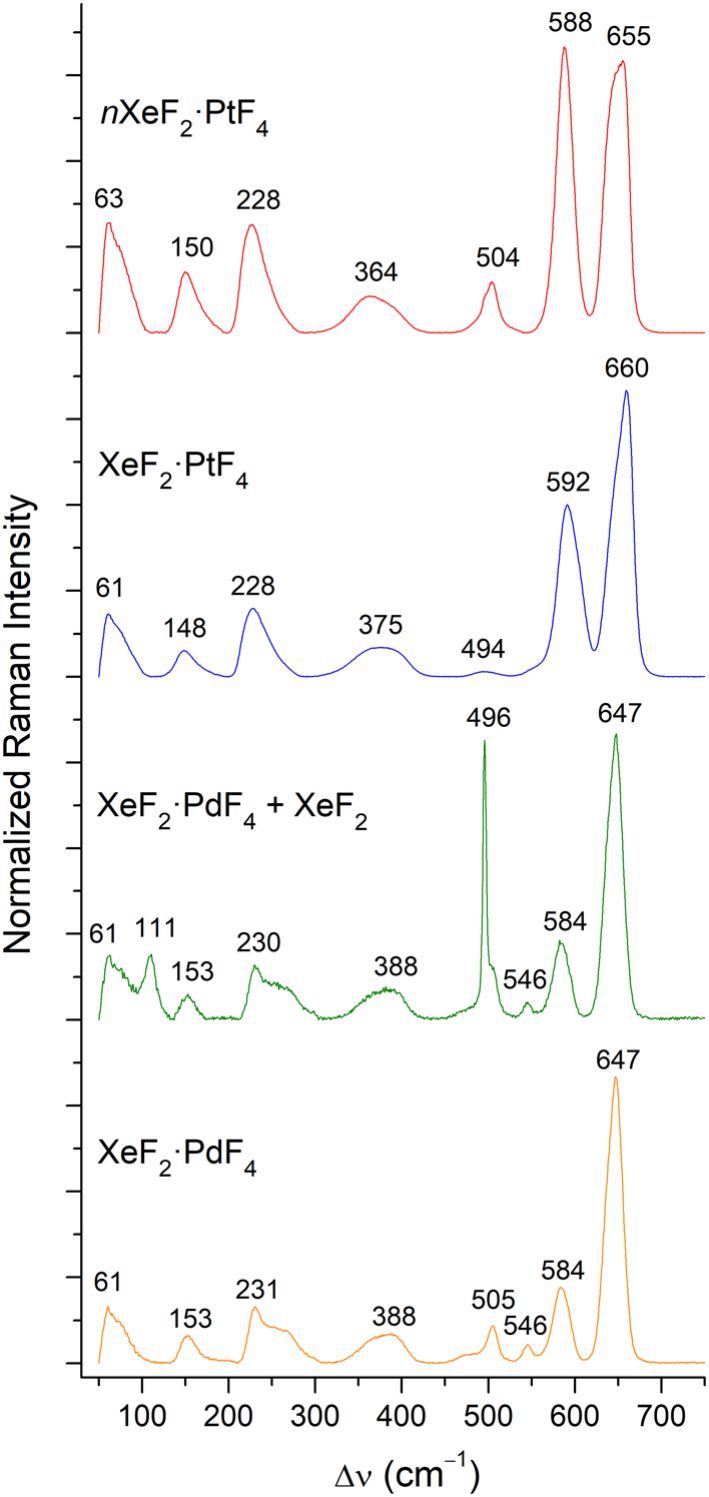
Ambient-temperature Raman spectra of the product of Pt oxidation by XeF_2_ in aHF, *n*XeF_2_·PtF_4_, *n* > 1 (red), the product of its pyrolysis under dynamic vacuum at 100 °C, XeF_2_·PtF_4_ (blue), mixture of XeF_2_·PdF_4_ and unreacted XeF_2_ (green), and XeF_2_·PdF_4_ (orange).

It is probable that the XeF_2_ “rich” phase *n*XeF_2_·PtF_4_ comprises cocrystallized XeF_2_ molecules, a structural feature analogous to what is observed in the crystal structure of 3XeF_2_·2MnF_4_.^19^ This is corroborated by a comparison of the Raman spectra of the compounds, as the ambient-temperature Raman spectrum of 3XeF_2_·2MnF_4_ exhibits a strong band at 506 cm^–1^ that has been assigned to cocrystallized XeF_2_ and thus corresponds well to the position of the band observed at 504 cm^–1^ in the Raman spectrum of *n*XeF_2_·PtF_4_ (*n* > 1) (Figure 3).

Previously, it was reported that the changes in the slope in a weight-loss curve indicated that the action of molten XeF_2_ on either PdF_4_ or Pd_2_F_6_ results in the formation of adducts *n*XeF_2_·PdF_4_ (*n* = 4, 3, 2, 1) all of which lose XeF_2_ under dynamic vacuum at room temperature except for XeF_2_·PdF_4_.^7^ The reinvestigation of the products of this reaction by Raman spectroscopy revealed that amorphous XeF_2_·PdF_4_, along with unreacted XeF_2_ (111, 496 cm^–1^), is the sole identifiable product (Figure 3), although the reaction product was not exposed to dynamic vacuum (eq 3). XeF_2_·PdF_4_ could be isolated from the product mixture by removing the excess XeF_2_ under dynamic vacuum at room temperature.

XeF2+PdF4→130C°XeF2·PdF4 3

The Raman spectrum of XeF_2_·PdF_4_ obtained during this study is in good agreement with the previously reported spectrum for this compound, which consisted of a strong band at 649 cm^–1^, accompanied by weaker lines at 585, 505, 385, and 230 cm^–1^,^7^ and shows a high degree of similarity to the Raman spectra of the platinum phases (Figure 3). The strong lines located at 588 and 592 cm^–1^ in the Raman spectra of the two platinum phases and the medium-strong line at 584 cm^–1^ in the spectrum of XeF_2_·PdF_4_ can be readily assigned to the Xe–F_t_ (t: terminal) stretching modes. The observed position of these bands is comparable to that observed in the ambient-temperature Raman spectrum of XeF_2_·MnF_4_, where two lines attributable to the Xe–F_t_ stretch are located at 580 and 587 cm^–1^,^19^ thereby indicating that a similar degree of XeF_2_ ionization is exhibited across this series of compounds. In comparison, the band attributable to the Xe–F_t_ stretch is located at 574 cm^–1^ in the Raman spectrum of XeF_2_·CrF_4_.^24^

Heating the XeF_2_·PtF_4_ and XeF_2_·PdF_4_ adducts above 150 °C under dynamic vacuum resulted in the release of XeF_2_ (eq 4). Powder X-ray diffraction (PXRD) patterns recorded on the resulting microcrystalline solids revealed them to be pure XeF_2_·2PtF_4_ and XeF_2_·2PdF_4_, respectively (Figures S1 and S2).

2(XeF2·MF4)→dynamicvacuum≥150C°XeF2·2MF4+XeF2(M=Pd,Pt) 4

Raman spectra of XeF_2_·2PtF_4_ and XeF_2_·2PdF_4_ were measured at a temperature of –173 °C (Figure 4 and Table S1) and exhibit excellent agreement with the previously reported ambient temperature spectra.^7^ In both spectra, the strongest band expected for the Xe–F_t_ stretching mode is observed at 609 cm^–1^. This value is comparable to the mode observed in other [XeF]^+^ compounds, such as [XeF]­[AsF_6_] (607, 611 cm^–1^)^10^ [XeF]­[BiF_6_] (602, 608 cm^–1^),^10^ and XeF_2_·2MnF_4_ (612 cm^–1^),^19^ and thus attests that the XeF_2_ ligand is highly ionized^22^ due to the substantial Lewis acidity of the 2MF_4_ unit or the low Lewis basicity of the [(M_2_F_9_)^−^]_∞_ backbone.

**Figure 4:**
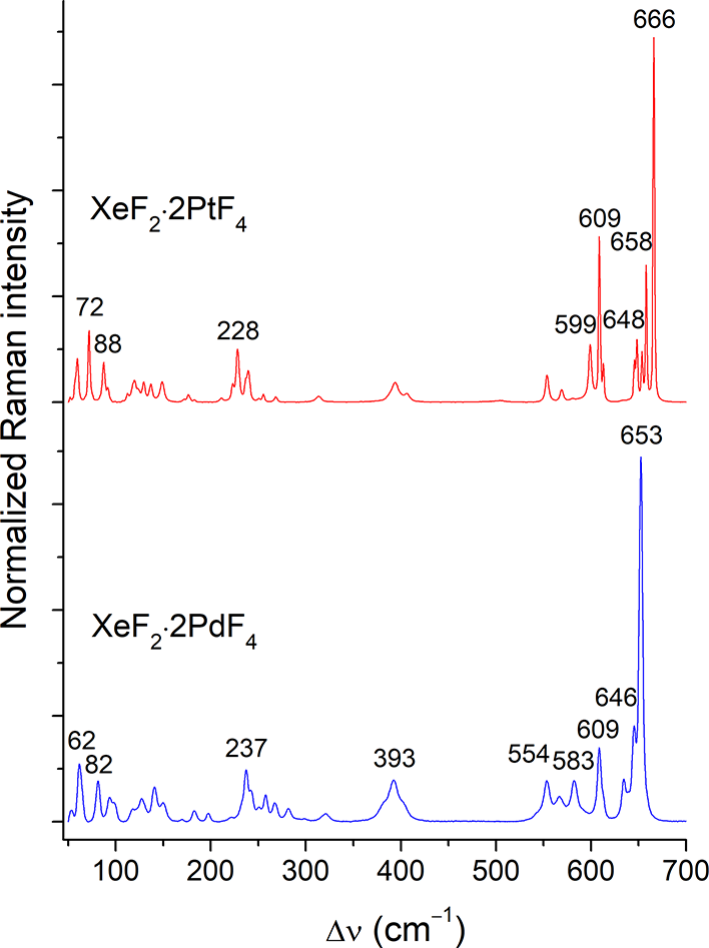
Low-temperature (−173 °C) Raman spectra of powdered XeF_2_·2PtF_4_ (top) and XeF_2_·2PdF_4_ (bottom).

### 2.2 Crystal Structures

The obtained powder samples of XeF_2_·2PtF_4_ and XeF_2_·2PdF_4_ were crystalline (Figures S1 and S2), however, the crystal sizes were too small for SCXRD analysis (Figure 2). Structural investigation by 3D ED revealed that XeF_2_·2PtF_4_ and XeF_2_·2PdF_4_ are isostructural and that this relationship extends also to the previously characterized XeF_2_·2MnF_4_.^19^ All compounds crystallize in the *P*2_1_/*n* space group with a slight increase in unit cell volume from 756.99(13) Å^3^ for XeF_2_·2MnF_4_ to 799.5(9) Å^3^ for XeF_2_·2PtF_4_ as the atomic number of the metal increases from Mn to Pt (Table 1).

**Table 1:** Summary of Crystal Data, Refinement Results, and Selected Bond Distances and Angles for the Structures of XeF_2_·2PtF_4_ and XeF_2_·2PdF_4_ Determined by 3D ED

	XeF_2_·2PtF_4_	XeF_2_·2PdF_4_
space group	*P*2_1_/*n*	*P*2_1_/*n*
*a* (Å)	5.4189(3)	5.3722(5)
*b* (Å)	9.8998(8)	9.959(3)
*c* (Å)	14.9993(9)	14.9091(8)
β (°)	96.506(5)	96.842(6)
*V* (Å^3^)	799.5(9)	792.0(2)
*Z*	4	4
*M*_w_ (g mol^–1^)	711.4	534
*D*_calcd_ (g cm^–3^)	5.9195	4.4788
*T* (K)	100	100
*R*_1_	0.0952	0.0657
w*R*_all_	0.1120	0.0685
Δ*V*_min_, Δ*V*_max_ (e Å^–1^)	–0.27, 0.29	–0.09, 0.10

Bond Lengths (Å) and Angles (°)		
Xe–F_t_	1.909(10)	1.892(5)
Xe–F_b_	2.316(8)	2.320(4)
M–F_b_(Xe)	1.932(8)	1.894(4)
M–F_b_(M)	1.957(7)–2.032(7)	1.946(4)–2.020(4)
M–F_t_	1.863(7)–1.905(11)	1.844(4)–1.866(4)
M–F–M	131.9(5)–133.0(4)	132.0(3)–134.4(3)
*cis*-F–M–F	86.2(4)–92.2(4)	87.0(3)–92.3(2)
*trans*-F–M–F	177.2(4)–179.5(4)	176.7(3)–179.3(2)
F–Xe–F	176.0(5)	178.0(3)

In both structures, the asymmetric unit comprises two crystallographically independent metal­(IV) centers, octahedrally coordinated by F atoms, with one of the centers coordinated by the XeF_2_ ligand. Both crystallographically independent [MF_6_] octahedra exhibit slightly distorted geometry, with the *cis*-F–M–F angles spanning a range of 86.2(4)–92.2(4)° in the structure of XeF_2_·2PtF_4_ and 87.0(3)–92.3(2)° in the case of XeF_2_·2PdF_4_ (Tables 1, S2 and S3). Each [MF_6_] unit shares three *fac*-F-vertices with three different neighboring octahedra, establishing a linkage whereby each XeF_2_-bearing octahedron is connected to three non-XeF_2_-carrying octahedra, and vice versa (Figures 5, S3 and S4). The bridging M–F_b_(M) bonds in XeF_2_·2PtF_4_ measure between 1.957(7) and 2.032(7) Å, whereas in XeF_2_·2PdF_4_ they range from 1.946(4) to 2.020(4) Å. The shorter M–F_t_ terminal bonds range from 1.863(7) to 1.905(11) Å in the case of XeF_2_·2PtF_4_ and from 1.844(4) to 1.866(4) Å in the XeF_2_·2PdF_4_ structure (Tables 1, S2 and S3).

**Figure 5:**
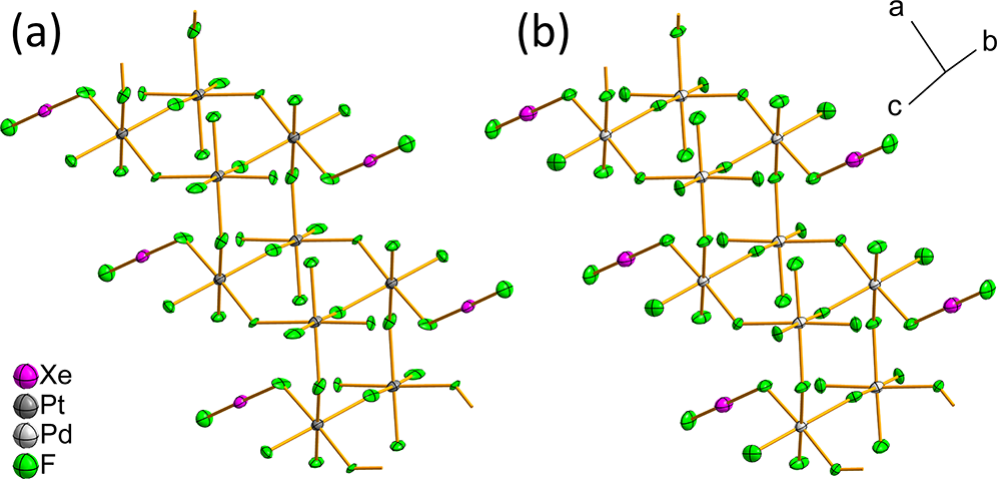
Sections of the crystal structures of (a) XeF_2_·2PtF_4_ and (b) XeF_2_·2PdF_4_ displaying the infinite corrugated zigzag double-chain motif. All atoms were refined anisotropically and are drawn as ellipsoids set at the 50% probability level.

This connectivity gives rise to infinite zigzag double chains propagating along the *a*-crystallographic axis, which can be formulated^25^ as _∞_^1^[⟨MF_2_F_3/1+1_(XeF_2_)⟩⟨MF_3_F_3/1+1_⟩] (Figure 5). In addition to the isostructural XeF_2_·2MnF_4_, analogous corrugated double-chain motifs have been previously observed in the [Ti_2_F_9_]^−^ anions, as evidenced in the crystal structures of, for example, Cs­[Ti_2_F_9_] and [ClO_2_]­[Ti_2_F_9_].^26,27^ Although oligo- or polymeric anions, which arise from interconnected [MF_6_] octahedra, have been observed to form with various tetravalent metals,^28^ XeF_2_·2PtF_4_ and XeF_2_·2PdF_4_ represent the first crystal structures featuring polymeric F-bridged fluoridoplatinates­(IV) and fluoridopalladates­(IV), respectively.

As previously established in the XeF_2_–TiF_4_ and XeF_2_–MnF_4_ systems,^13,19^ the increased dimensionality of the polyfluoridometallate backbone also leads to an increase in Lewis acidity, which is evidenced by the substantial ionization of the coordinated XeF_2_ molecule. The bridging Xe–F_b_ bonds are highly elongated, measuring 2.316(8) and 2.320(4) Å in the structures of XeF_2_·2PtF_4_ and XeF_2_·2PdF_4_, respectively, whereas the Xe–F_t_ terminal bonds are contracted, measuring 1.909(10) and 1.892(5) Å, respectively. In the crystal structure of XeF_2_ (*D*_∞h_), the Xe–F bond distance was determined to be 1.999(4) Å by SCXRD^10^ and 1.992(8) Å by 3D ED.^29^ The Xe–F_b_ bond length exceeding 2.3 Å has been hitherto observed exclusively in the crystal structure of [XeF]^+^[Sb_2_F_11_]^−^, which forms if XeF_2_ is reacted with an excess of Lewis superacid SbF_5_.^10,30^ This attests to the unprecedented Lewis acidity exhibited by the chains and confirms that the two XeF_2_·2MF_4_ compounds can be regarded as a tight-ion pair salts, [XeF]^+^[M_2_F_9_]^−^.

Additionally, the crystal structures are stabilized by 8 Xe···F contacts, shorter than the corresponding sum of the van der Waals radii (3.52 Å),^31^ with 3 of the contacts formed by F atoms, which are part of the same chain as the coordinated XeF_2_ moiety and 5 belonging to the neighboring chains (Figures S5 and S6). The Xe···F contacts measure between 3.019(10) and 3.421(10) Å in the case of XeF_2_·2PtF_4_ and between 3.049(6) and 3.427(7) Å in the case of XeF_2_·2PdF_4_ (Tables S4 and S5).

### 2.3 Structural Models of XePtF_6_

Computational evaluation of the structural models of XeF_2_·PtF_4_ was performed using periodic density functional theory calculations, utilizing r^2^SCAN meta-GGA functional supplemented with the D3 van der Waals corrections. The computational approach was initially benchmarked by performing geometry optimization calculations for XeF_2_·CrF_4_ and XeF_2_·MnF_4_ in the three considered structure types: XeF_2_·CrF_4_,^15^ XeF_2_·MnF_4_,^19^ and 3XeF_2_·2MnF_4_ with cocrystallized XeF_2_ removed. For the sake of clarity, each of these structures will henceforth be labeled with respect to the connectivity of the [MF_6_] units: *trans*-chains (= XeF_2_·CrF_4_), *cis*-chains (= 3XeF_2_·2MnF_4_; cocrystallized XeF_2_ removed), and tetramers (= XeF_2_·MnF_4_).

For XeF_2_·CrF_4,_ the results show that the *trans*-chain geometry has the lowest energy, in accordance with the experimentally observed structure (Figure 1).^15^ The tetrameric ring polymorph is higher in energy (+9.8 kJ/mol per formula unit), whereas the *cis*-chain structure is even more destabilized (+11.6 kJ/mol). Calculations suggest that the lowest magnetic state for *trans*-chain XeF_2_·CrF_4_ is an antiferromagnetic (AFM) arrangement of the unpaired electrons along the chains, which is in agreement with the previously reported magnetic measurements.^32^ For XeF_2_·MnF_4,_ the experimental tetrameric structure has the lowest energy with *cis*- and *trans*-chains higher in energy (+3.2 and +16.3 kJ/mol, respectively). An AFM coupling of the magnetic moments within the tetramers is found as the lowest magnetic configuration. For both XeF_2_·CrF_4_ and XeF_2_·MnF_4_, the DFT-derived geometries of the lowest-energy structures are in close agreement with the experimental ones (Tables S6 and S7). In addition, geometry optimization calculations were also performed for XeF_2_·2PtF_4_ and XeF_2_·2PdF_4_, with the resulting bond lengths and angles showing good agreement with the experimentally determined structures (Tables S2 and S3).

Calculations performed for XeF_2_·PtF_4_ (Figure 6 and Table 2) yield the same structure preferences as in the case of XeF_2_·MnF_4_: the tetrameric ring (XeF_2_·MnF_4_-type) structure has the lowest energy, with the *cis*-chain polymorph (3XeF_2_·2MnF_4_-type; cocrystallized XeF_2_ removed) slightly higher in energy (+3.7 kJ/mol), and the *trans*-chain structure (XeF_2_·CrF_4_-type) much less stable by a significant value (+22.5 kJ/mol). These results indicate that the hypothesized *trans*-bridged chain structure based on XeF_2_·CrF_4_,^15^ which has been previously proposed as a model for the structure of XeF_2_·PtF_4_,^4^ is improbable, with *cis*-bridging among the neighboring octahedra, either in the form of tetramers or infinite chains, evidently preferred. Similar trend was observed in calculations performed for XeF_2_·PdF_4_, with the tetrameric structure proving to be the most energetically favorable, followed by *cis*-chain (+4.6 kJ/mol) and *trans*-chain (+22.7 kJ/mol) structures. Additional probing of the potential energy surface of solid XeF_2_·PtF_4_ was performed using an evolutionary algorithm search, which yielded 400 structures whose energy and geometry were optimized by DFT calculations. None of the generated structures had a lower energy than the tetrameric ring model, corroborating the notion that this is a likely ground-state structure of XeF_2_·PtF_4_, at least at ambient pressure (*P* = 1 atm).

**Figure 6:**
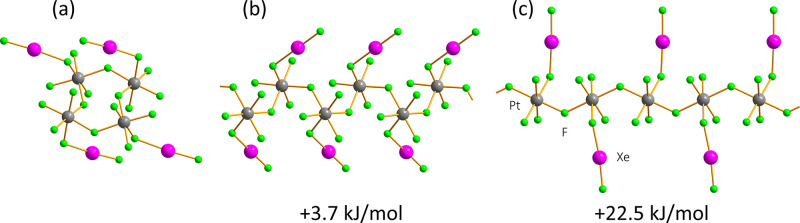
Periodic DFT-optimized structural models of XeF_2_·PtF_4_ (XePtF_6_) and the differences in their relative stabilities: (a) tetrameric ring, (b) *cis*-chain, and (c) *trans*-chain.

**Table 2:** DFT-Calculated Unit-Cell Parameters and Selected Bond Distances and Angles for the Three Candidate Structures of XeF_2_·PtF_4_

	XeF_2_·PtF_4_ (tetrameric rings)	XeF_2_·PtF_4_ (*cis*-chains)	XeF_2_·PtF_4_ (*trans*-chains)
Space group	*P*2_1_/*n*	*P*2_1_/*n*	*P*2_1_/*n*
*a* (Å)	9.630	9.794	7.425
*b* (Å)	11.151	5.038	7.215
*c* (Å)	9.872	11.457	10.032
β (°)	93.76	94.91	92.78
*V* (Å^3^)	1057.75	563.32	536.78
*Z*	8	4	4

Bond Lengths (Å), Angles (°)			
Xe–F_t_	1.981, 1.988	1.989	1.983
Xe–F_b_	2.241, 2.259	2.230	2.242
M–F_b_(Xe)	2.004, 2.022	2.009	2.004
M–F_b_(M)	2.018–2.032	2.019, 2.019	1.977, 1.980
M–F_t_	1.897–1.907	1.890–1.904	1.897–1.940
M–F–M	130.89, 131.78	134.04	131.47
*cis*-F–M–F	87.12–94.08	83.85–95.90	84.33–95.80
*trans*-F–M–F	175.58–179.84	175.71–177.22	178.30–178.90
F–Xe–F	174.83, 175.24	174.95	173.33

These results testify to the similarity in the structural landscape of the studied XeF_2_–PtF_4_ and XeF_2_–MnF_4_ compounds, in particular the preference for the formation of *cis*-bridges between adjacent metal centers. The calculations predict that in all of the investigated XeF_2_·PtF_4_ polymorphs the Pt^IV^ (d^6^ electron count) adopt a low-spin configuration resulting in no net magnetic moment. This is in accordance with previous suggestions that pure XePtF_6_ is diamagnetic, as the weak paramagnetic response of samples of this compound were the result of contamination with Pt^V^ compounds.^4^

## 3 Conclusions

In the present work, the chemistry of the XeF_2_–PtF_4_ and XeF_2_–PdF_4_ systems was revisited. The interaction of XeF_2_ with metallic Pt in aHF solvent yielded an orange-red solution from which *n*XeF_2_·PtF_4_ (1 < *n* < 1.6) was isolated, and its Raman spectrum indicated that it contains cocrystallized XeF_2_. The pyrolysis of this compound at 100 °C under dynamic vacuum resulted in the release of the cocrystallized XeF_2_ giving rise to an orange solid, whose composition is likely XeF_2_·PtF_4_. The Raman spectra of both solids also exhibited a high degree of similarity to the Raman spectrum of XeF_2_·PdF_4_, which was prepared by the interaction of Pd_2_F_6_/PdF_4_ mixture with molten XeF_2_. However, the detailed structural investigation of these compounds was precluded due to their invariably amorphous nature. When exposed to dynamic vacuum at temperatures of 160 and 150 °C, respectively, XeF_2_·PtF_4_ and XeF_2_·PdF_4_ undergo pyrolysis, releasing XeF_2_ and resulting in the formation of microcrystalline XeF_2_·2PtF_4_ and XeF_2_·2PdF_4_. Their structures were successfully elucidated by 3D ED, making them the first structurally characterized XeF_2_–MF_4_ (M = Pd, Pt) compounds and revealing them to be isostructural with the previously investigated XeF_2_·2MnF_4_.^19^ This observation provides the first evidence that directly links the XeF_2_–PtF_4_ system with the structurally well-characterized XeF_2_–MnF_4_ compounds. Moreover, XeF_2_·2PtF_4_ and XeF_2_·2PdF_4_ exhibit nearly identical PXRD patterns, corroborating their isostructural nature and the full profile fits using the Rietveld method also confirmed the structures derived from 3D ED.

In addition, quantum chemical calculations were utilized to assess the relative stabilities of XeF_2_·PtF_4_ in three distinct structural types, which were derived from the experimentally determined structures. The results indicated that the tetrameric-ring structure, based on XeF_2_·MnF_4_, is the most stable and that *cis*-bridging among the adjacent [PtF_6_] units is favored. The computational approach was benchmarked by performing the optimizations also for XeF_2_·CrF_4_ and XeF_2_·MnF_4_ in all three evaluated structural types, yielding the experimentally observed structure as the lowest in energy in both cases. The combined identification of isostructural relationships across the XeF_2_–PtF_4_ and XeF_2_–MnF_4_ systems, with the observed highest relative stability of the tetrameric ring-based structure closely followed by a *cis*-chain, establishes the XeF_2_·MnF_4_-type structures as the leading candidates for the structure of the first noble-gas compound XeF_2_·PtF_4_ (XePtF_6_).

## 4 Experimental Section

**Caution:** The handling of anhydrous HF, F_2_, and high-valent fluorides requires great care and should be conducted only by appropriately trained experimentalists in a well-ventilated fume hood. It is imperative that protective equipment is worn at all times, and that access to proper treatment procedures is available in case of exposure.^33,34^

### 4.1 Materials and Methods

#### 4.1.1 Reagents and Apparatus

F_2_ (Solvay Fluor, 98–99%), LiF (Aldrich, ≥99.98%), Pd (Sigma-Aldrich, 99.9%), and Pt powder (Thermo Scientific, 99.98%) were used as supplied without further purification. Prior to its use, commercial anhydrous HF (aHF; Linde, 99.995%) was condensed in a FEP reactor vessel loaded with K_2_NiF_6_ (Advance Research Chemicals, 99.9%) and left to stir overnight, thus eliminating the residual traces of moisture.^4,35^ XeF_2_ was synthesized via a gas-phase UV-photochemical reaction of F_2_ and Xe, as previously described.^36^ AsF_5_ was prepared by the static fluorination of As_2_O_3_ in a nickel reaction vessel.^37^

All xenon­(II) compounds examined in this study are highly moisture sensitive and require that all experimental procedures be conducted under strictly anhydrous conditions. The dispensing of volatiles and the work with aHF solvent were conducted on a custom-built vacuum line constructed from nickel, copper, polytetrafluoroethylene (PTFE) and fluorinated ethylene propylene (FEP). The handling of solids was conducted in a glovebox (Vigor SG1200/750E–SG1500/750E) containing an inert nitrogen atmosphere, with moisture levels maintained below 1 ppm at all times.

Thermal syntheses were conducted in PTFE-gasketed nickel reactors. Reactions in aHF were performed in reactors constructed of 16 mm i.d. × 19 mm o.d. FEP tubing, capped with brass-encased PTFE valves. Prior to use, all reactors were passivated with 300–700 Torr of F_2_ overnight.

#### 4.1.2 Synthesis of nXeF_2_·PtF_4_ (*n* > 1), XeF_2_·PtF_4_, and XeF_2_·2PtF_4_

The first step of the synthesis involved the oxidation of Pt powder by an excess of XeF_2_ in aHF solvent, following the previously outlined approach.^23^ In an inert atmosphere glovebox, 327 mg (1.68 mmol) of platinum powder and 1020 mg (6.03 mmol) of XeF_2_ were loaded into a reactor constructed from a 16 mm i.d. FEP tubing (*V* ≈ 32 mL). The reactor was connected to the vacuum line and 2.4 mL of aHF was condensed on the solid reagents at the temperature of –196 °C. After the reactor contents were warmed to room temperature and the resulting mixture agitated, a gradual change in the color of the solution from colorless to orange-red was observed. Following an additional 16 h of agitation, an orange-red solution above a viscous dark-red liquid phase was observed in the reactor (Figure 2). The products were isolated by removing the volatiles under a dynamic vacuum at temperatures between −40 and −60 °C, resulting in the formation of an orange-red solid together with colorless crystals. The residual material was homogenized in a mortar and filled into a quartz capillary for analysis by PXRD and Raman spectroscopy. PXRD measurements revealed predominantly diffuse scattering, accompanied by very weak lines that could be attributed to XeF_2_, whereas the Raman spectrum (Figure 3, top) resembled those previously reported for XeF_2_·PtF_4_ and XeF_2_·PdF_4_,^4,7^ with a peak at 504 cm^–1^ and a shoulder at approximately 496 cm^–1^ likely corresponding to the cocrystallized and admixed XeF_2_, respectively. Subsequently, 486 mg of the resulting product mixture was transferred to a nickel reaction vessel (*V* ≈ 7.4 mL) which was then connected to the vacuum line via a U-shaped trap constructed of 6 mm i.d. FEP tubing (Figure S7). The reactor was heated to a temperature of 100 °C while held under dynamic vacuum and the released XeF_2_ was collected in the FEP trap, which was cooled by liquid nitrogen. After 5 h, the accumulation of XeF_2_ in the trap ceased. The orange material XeF_2_·PtF_4_ remaining in the reactor was loaded into a quartz capillary for analysis, which revealed no lines in the PXRD pattern (Figure S1) and yielded a Raman spectrum that was very similar to that of the initially formed orange-red product, albeit without the signals at 496/504 cm^–1^ (Figure 3, middle). A further round of pyrolysis under a dynamic vacuum was conducted at 160 °C. After 3.5 h, the accumulation of XeF_2_ ceased. The PXRD pattern (Figures S1 and S2) and Raman spectrum (Figure 4) of the resulting peach-colored crystalline material (Figure 2) indicated that pure XeF_2_·2PtF_4_ had formed.^7^

#### 4.1.3 Synthesis of XeF_2_·PdF_4_ and XeF_2_·2PdF_4_

An attempt was made to synthesize PdF_4_ following the previously described route by displacing it from Li_2_PdF_6_ dissolved in aHF through the use of AsF_5_.^4^ Li_2_PdF_6_ was prepared by oxidizing Pd powder with F_2_ in the presence of LiF in aHF solvent.^38^ In all attempts to synthesize PdF_4_ using this procedure, the resulting PdF_4_ was partially reduced, forming a mixture of Pd_2_F_6_ and PdF_4_.

In a typical experiment, a nickel reaction vessel (*V* ≈ 7.4 mL) was loaded with XeF_2_ (522 mg, 3.08 mmol) and the Pd_2_F_6_/PdF_4_ mixture (61 mg) inside an inert atmosphere glovebox. The sealed reactor was transferred to an oven, which had been preheated to 130 °C, where it was left for 16 h. This resulted in a mixture of yellow solid XeF_2_·PdF_4_ and colorless XeF_2_ crystals as revealed by Raman spectroscopy (Figure 3). Unreacted XeF_2_ can be removed from the product by sublimation under dynamic vacuum at room temperature. The produced pure XeF_2_·PdF_4_ is amorphous and exhibits broad Raman bands that are in accordance with the previously reported results (Figure 3).^7^ The pyrolysis of XeF_2_·PdF_4_ was conducted under dynamic vacuum at a temperature of 150 °C,^7^ resulting in the release of XeF_2_, which was collected in a U-trap cooled with liquid nitrogen. The decomposition was complete after 5 h, yielding an orange-brown crystalline agglomerated powder (Figure 2) of pure XeF_2_·2PdF_4_, as revealed by PXRD (Figure S2) and Raman spectroscopy (Figure 4).^7^

### 4.2 Characterization

#### 4.2.1 Powder X-ray Diffraction (PXRD)

Powder X-ray diffraction patterns were obtained on a Rigaku OD XtaLAB Synergy-S diffractometer equipped with a Dectris EIGER2 R CdTe 1M detector using microfocused Ag Kα radiation (λ = 0.56087 Å). PXRD profiles were extracted and processed using *CrysAlisPro* software.^39^ The measurements were conducted at 100 K, using an Oxford Cryosystems 800 Series Cryostream. The measurement parameters employed in this study were consistent with those previously reported.^19,40^ The samples were pulverized in an agate mortar within a glovebox and loaded into quartz capillaries, which were subsequently flame-sealed in a hydrogen–oxygen flame. Prior to use, quartz capillaries (500 μm diameter) were thoroughly dried under dynamic vacuum and passivated with F_2_. *GSAS-II* program^41^ was used for the structural refinements and quantitative phase analyses with the Rietveld refinement method.^42^ Crystal structures determined by 3D ED were used to fit the PXRD data (Figure S2).

#### 4.2.2 Vibrational Spectroscopy

Ambient and low-temperature Raman spectra were measured on a Bruker Senterra II confocal Raman microscope using a 785 nm emission line. The spectra were measured in the 50–1410 cm^–1^ range with a resolution of 1.5 cm^–1^. An aperture of 50 × 1000 μm was used to acquire the spectra of XeF_2_·PdF_4_, XeF_2_·PdF_4_ with admixed XeF_2_, XeF_2_·PtF_4_ and *n*XeF_2_·PtF_4_, whereas the spectra of XeF_2_·2PtF_4_ and XeF_2_·2PdF_4_ were obtained using an aperture of 15 × 1000 μm. The nominal power of the laser used was varied between 100 mW (XeF_2_·2PtF_4_), 25 mW (XeF_2_·2PdF_4_ XeF_2_·PtF_4_, *n*XeF_2_·PtF_4_), and 10 mW (XeF_2_·PdF_4_, XeF_2_·PdF_4_ with admixed XeF_2_). Low-temperature spectra were measured at −173 °C employing a Linkam LTS420 low-temperature stage. Sample preparation involved loading the materials into quartz capillaries, as described in Section 4.2.1. Background subtraction from the spectra was performed using the Bruker *OPUS* 8.7 software suite, with 3 iterations of concave rubber band correction and 64 baseline points.

Infrared spectra (Figures S8 and S9) were measured inside an N_2_ atmosphere glovebox on a Bruker Alpha II FT-IR spectrometer equipped with a Platinum Diamond-ATR sampling module. Prior to analysis, the compounds were finely ground in an agate mortar. Spectra were recorded in the range of 400–4000 cm^–1^ with a resolution of 4 cm^–1^ and 24 scans were taken per measurement.

#### 4.2.3 3D Electron Diffraction

3D ED data collection was performed on an FEI Tecnai G^2^ transmission electron microscope (TEM). Data sets were collected at a temperature of 100 K using a continuous rotation electron diffraction (cRED) method with a tilt step of 0.30° per diffraction frame. The electron beam was generated using a LaB_6_ source at 200 kV (λ = 0.02508 Å), and a Medipix 3 hybrid pixel detector ASI CheeTah (512 × 512 pixels, 24-bit dynamic range) was used to collect the diffraction patterns.

Recently, a novel cryotransfer protocol was developed and successfully applied for the study of several air-sensitive and highly oxidizing xenon compounds, including XeF_2_·2MnF_4_.^19^ Following this procedure,^29^ the samples were transferred directly from the FEP vessels in an N_2_-filled glovebox to the cooled (< −150 °C) cryo-holder (Gatan Model 914), ensuring an air- and moisture-free environment. Then, the cooled cryo-holder was quickly transferred into the TEM.

The crystallites of XeF_2_·2PtF_4_ and XeF_2_·2PdF_4_, from which the 3D ED data were collected, are shown in Figures 2, S10 and S11. In the case of XeF_2_·2PtF_4_, the data were collected on two crystals and subsequently merged. The diffraction data were processed in *PETS2* software^43^ for indexation, lattice parameter determination, and data reduction. The optical distortions of the diffraction patterns were taken into account to ensure accurate lattice parameter determinations.^44^ Then the processed data were imported into *JANA2020* ^45^ for crystal structure determination ab initio using *SIR2014*.^46^ The structures were first refined kinematically and then, without further optimization of kinematical refinement, they were refined dynamically.^47^ For the dynamical calculation of the diffracted intensities, an incoherent mosaicity model was used, which assumes the crystals to be composed of large mosaic blocks that span the entire crystal thickness.^48^ Including this model significantly improved the *R*-factors by about 3.8–4.8%. Further information on the experimental setup for 3D ED can be found in the SI (Tables S8–S10). Molecular graphics were prepared using the *Diamond* 5.0 software.^49^

### 4.3 Periodic DFT Calculations

Periodic plane-wave density functional theory (DFT) calculations were performed within the projector-augmented-wave (PAW) method as implemented in *VASP* 6.4.3.^50,51^ r^2^SCAN meta-GGA functional^52,53^ supplemented with the D3 van der Waals corrections^54,55^ was employed, which has proven to yield more accurate results for the modeling of palladium fluorides than the GGA-based PBE functional.^56^ The calculations were performed using a plane-wave basis set with a cutoff energy of 700 eV, and the convergence criterion for electronic minimization was 10^–6^ eV. Brillouin zone sampling was performed using the Monkhorst–Pack mesh with a *k*-point spacing of 2π × 0.033 Å^–1^.^57^ Geometry optimization of the crystal structures was conducted with all free parameters (i.e., those not constrained by space group symmetry) allowed to vary. The optimization was conducted until the forces acting on atoms were smaller than 5 meV/Å and stresses were below 100 bar. Calculations were conducted assuming spin-polarization of the Kohn–Sham orbitals, and for each structure and stoichiometry, several spin states were tested—the geometry optimization was performed for the one exhibiting the lowest energy.

The optimization was conducted for XeF_2_·2PtF_4_, XeF_2_·2PdF_4_, and XeF_2_·MF_4_ (M = Cr, Mn, Pd, Pt) compounds. For the latter, three possible structure types were considered: XeF_2_·CrF_4_,^15^ XeF_2_·MnF_4_,^19^ and a structure derived from 3XeF_2_·2MnF_4_ ^19^ by removing the cocrystallized XeF_2_ molecules (yielding a XeF_2_·MF_4_ stoichiometry). The energies (per formula unit) of the optimized structures were compared as a proxy for their relative stability. Atom coordinates of the DFT-calculated crystal structures are reported in the SI (Table S11).

Evolutionary structure searches for structure candidates for XeF_2_·PtF_4_ were conducted in the *XtalOpt* program (version r12).^58^ The searches for crystal structures containing up to 4 XePtF_6_ formula units in the primitive unit cell were carried out at ambient pressure at the same level of theory as described above.

## Supplementary Material



## Data Availability

The data supporting the findings of this study are available on the Zenodo open repository https://zenodo.org/ under the DOI number: 10.5281/zenodo.15594028.

## Notes

PXRD data and Rietveld refinement results, tabulated Raman data, additional crystallographic data and figures displaying crystal packing and nonbonded Xe···F contacts, comparison of the experimental and DFT-calculated structural parameters, ATR-IR spectra, and the coordinates of the DFT-calculated structures (PDF)
