# Supplementary material for: Crystal Structures of XeF2·2PtF4 and XeF2·2PdF4 Determined by 3D Electron Diffraction and Structural Models of XePtF6
Source: Inorg Chem. 2025 Jul 14;64(29):14968–76. doi: 10.1021/acs.inorgchem.5c01740 (PMC12308788; doi:10.1021/acs.inorgchem.5c01740)
Supplement: Supplementary file 1 [file ic5c01740_si_001.pdf]

Supporting Information

Crystal Structures of $XeF_2 \cdot 2PdF_4$ and $XeF_2 \cdot 2PtF_4$ Determined by 3D Electron Diffraction and Structural Models of $XePtF_6$

Klemen Motaln,[1,2] Kshitij Gurung,[3] Mirela Dragomir,[1,2] Dominik Kurzydłowski,[4] Lukáš Palatinus,[3*] and Matic Lozinšek[1,2*]*

[1] *Jožef Stefan Institute, Jamova cesta 39, 1000 Ljubljana, Slovenia*

[2] *Jožef Stefan International Postgraduate School, Jamova cesta 39, 1000 Ljubljana, Slovenia*

[3] *Department of Structure Analysis, Institute of Physics of the Czech Academy of Sciences, Na Slovance 1999/2, Prague 8, 18221, Czech Republic*

[4] *Faculty of Mathematics and Natural Sciences, Cardinal Stefan Wyszyński University in Warsaw, ul. Wóycickiego 1/3, 01-938 Warsaw, Poland*

Corresponding authors' email: matic.lozinsek@ijs.si (M.L.), palat@fzu.cz (L.P.), d.kurzydlowski@uksw.edu.pl (D.K.)

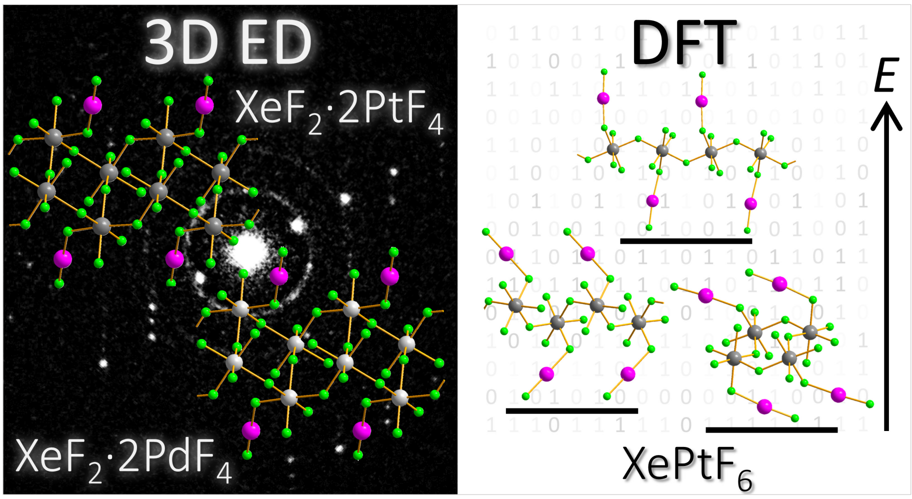

DOI: 10.1021/acs.inorgchem.5c01740

Table of Contents

Figure S1. PXRD frames of the amorphous $XeF_2{\cdot}PtF_4$ phase and the crystalline $XeF_2{\cdot}2PtF_4$ S3

Figure S2. Qualitative comparison of the laboratory powder X-ray diffraction patterns (100 K) of $XeF_2{\cdot}2PtF_4$ and $XeF_2{\cdot}2PdF_4$ and Rietveld refinement results for $XeF_2{\cdot}2PtF_4$ and $XeF_2{\cdot}2PdF_4$ S4

Table S1. Observed bands in the low-temperature Raman spectra of $XeF_2{\cdot}2PtF_4$ and $XeF_2{\cdot}2PdF_4$ S5

Table S2. Experimental (3D ED) and calculated (r^2SCAN-D3) geometrical parameters for $XeF_2{\cdot}2PtF_4$ S6

Table S3. Experimental (3D ED) and calculated (r^2SCAN-D3) geometrical parameters for $XeF_2{\cdot}2PdF_4$ S7

Figure S3. The crystal packing and the unit cell of $XeF_2{\cdot}2PtF_4$ S8

Figure S4. The crystal packing and the unit cell of $XeF_2{\cdot}2PdF_4$ S9

Figure S5. Xe1 coordination environment in the crystal structure of $XeF_2{\cdot}2PtF_4$ S10

Table S4. Non-bonded Xe···F contacts in the crystal structure of $XeF_2{\cdot}2PtF_4$ S10

Figure S6. Xe1 coordination environment in the crystal structure of $XeF_2{\cdot}2PdF_4$ S11

Table S5. Non-bonded Xe···F contacts in the crystal structure of $XeF_2{\cdot}2PdF_4$ S11

Table S6. Experimental (SCXRD) and calculated (r^2SCAN-D3) unit-cell and geometrical parameters for $XeF_2{\cdot}CrF_4$ S12

Table S7. Experimental (SCXRD) and calculated (r^2SCAN-D3) geometrical parameters for $XeF_2{\cdot}MnF_4$ S13

Figure S7. Experimental setup used for the pyrolysis of the XeF_2–PtF_4 and XeF_2–PdF_4 adducts S14

Figure S8. ATR-IR spectra $nXeF_2{\cdot}PtF_4$ (n > 1), $XeF_2{\cdot}PtF_4$, and $XeF_2{\cdot}PdF_4$ S15

Figure S9. ATR-IR spectra of $XeF_2{\cdot}2PtF_4$ and $XeF_2{\cdot}2PdF_4$ S15

Figure S10. TEM images of $XeF_2{\cdot}2PtF_4$ crystallites that were used for the 3D ED experiment S16

Table S8. 3D ED data collection and results for $XeF_2{\cdot}2PtF_4$ S16

Figure S11. TEM image of $XeF_2{\cdot}2PdF_4$ crystallite used for 3D ED experiment S17

Table S9. 3D ED data collection and results for $XeF_2{\cdot}2PdF_4$ S17

Table S10. General microscope information S18

Table S11. Coordinates of the DFT-calculated (r^2SCAN-D3) crystal structures S18

References S24

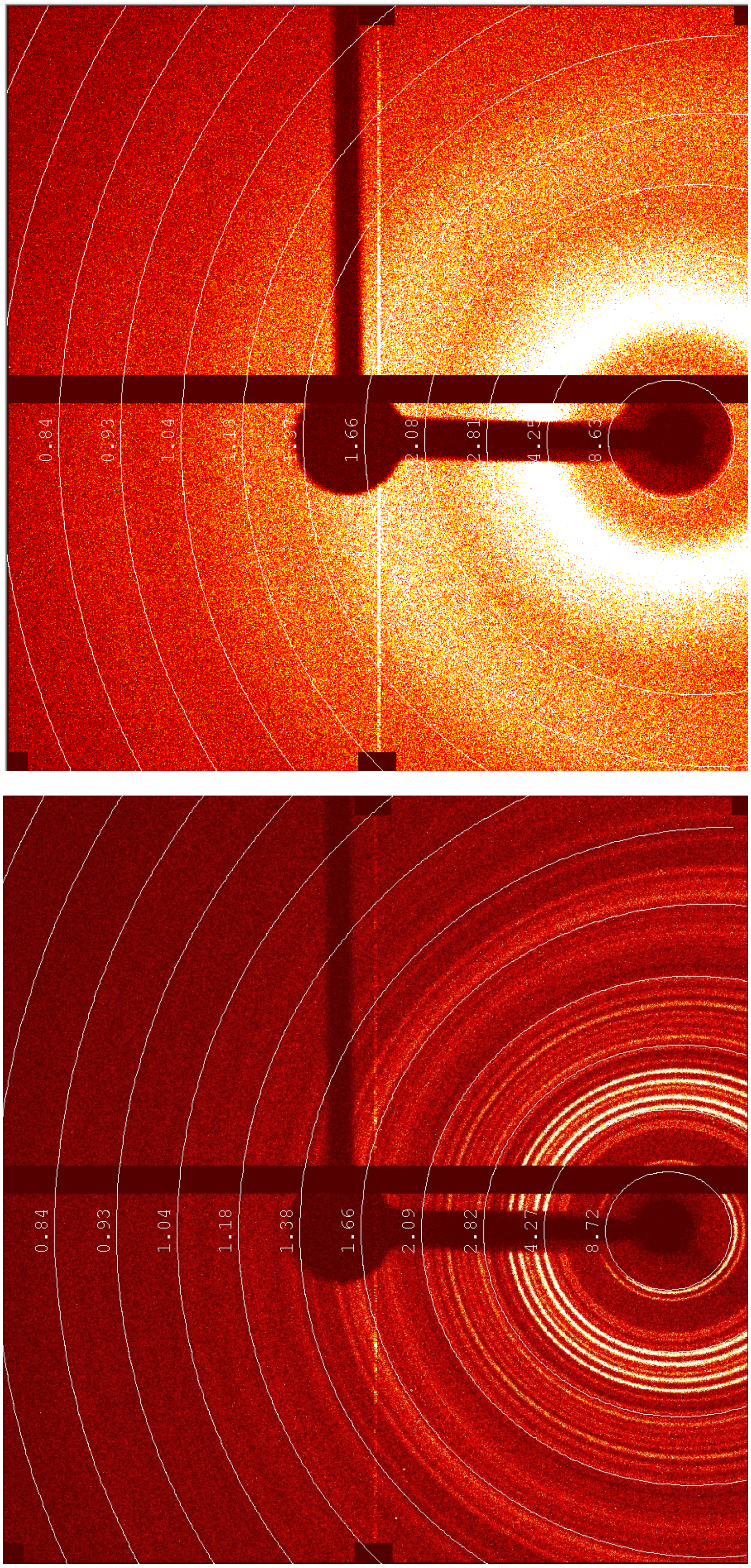

Figure S1. PXRD frames of the amorphous $XeF_2 \cdot PtF_4$ phase obtained by pyrolysis at 100 °C (top) and the crystalline $XeF_2 \cdot 2PtF_4$ obtained by pyrolysis at 160 °C (bottom).

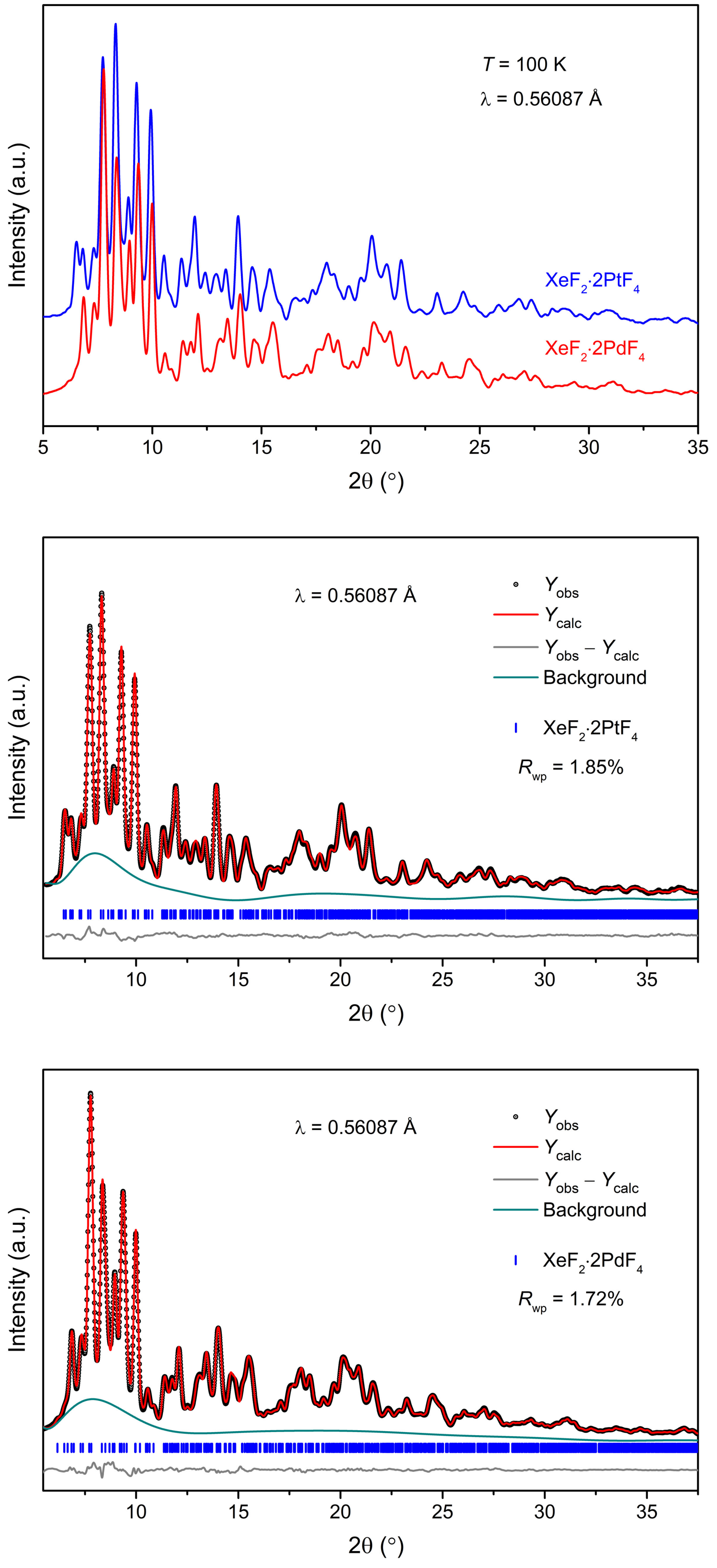

Figure S2. Qualitative comparison of the laboratory powder X-ray diffraction patterns (100 K) of $XeF_2{\cdot}2PtF_4$ and $XeF_2{\cdot}2PdF_4$ (top) and Rietveld refinement results for $XeF_2{\cdot}2PtF_4$ (middle) [$P2_1/n$, a = 5.4155(5) Å, b = 9.9161(9) Å, c = 14.9441(21) Å, β = 96.636(5)°, V = 797.14(15) Å^3; R_{wp} = 1.85%; χ^2 = 1.61] and $XeF_2{\cdot}2PdF_4$ (bottom) [$P2_1/n$, a = 5.3560(3) Å, b = 9.8822(5) Å, c = 14.8523(14) Å, β = 97.006(3)°, V = 780.24(8) Å^3, R_{wp} = 1.72%, χ^2 = 2.73].

Table S1. Observed bands in the low-temperature Raman spectra of $XeF_2{\cdot}2PtF_4$ and $XeF_2{\cdot}2PdF_4$ (Figure 4) with their relative intensities denoted in parentheses.

$XeF_2{\cdot}2PtF_4$ Δν (cm^{-1})	$XeF_2{\cdot}2PdF_4$ Δν (cm^{-1})
52 (1)	54 (3)
60 (12)	62 (16)
72 (20)	82 (11)
88 (11)	94 (7)
92 (4)	99 (5)
112 (2)	118 (3)
120 (6)	128 (6)
130 (6)	141 (9)
137 (5)	150 (5)
149 (6)	183 (3)
177 (2)	198 (2)
212 (1)	237 (14)
223 (5)	242 (9)
228 (15)	251 (4)
240 (9)	258 (7)
256 (2)	267 (5)
269 (2)	282 (4)
314 (2)	321 (2)
394 (5)	393 (11)
407 (2)	554 (11)
554 (7)	567 (7)
569 (3)	583 (11)
599 (16)	609 (20)
609 (45)	635 (12)
613 (10)	646 (26)
645 (12)	653 (100)
648 (17)	
654 (14)	
658 (38)	
666 (100)	

Table S2. Experimental (3D ED) and calculated (r^2SCAN-D3) geometrical parameters for $XeF_2{\cdot}2PtF_4$

Bond distance (Å)	Experimental	Calculated	Bond distance (Å)	Experimental	Calculated
Xe1–F1	1.909(10)	1.969	Pt2–F3	1.992(13)	2.028
Xe1–F2	2.316(8)	2.313	Pt2–F4	2.014(8)	2.025
Pt1–F2	1.932(8)	1.950	Pt2–F5^{ii}	2.032(7)	2.035
Pt1–F3	2.023(12)	2.022	Pt2–F8	1.888(14)	1.898
Pt1–F4^{i}	1.984(9)	2.009	Pt2–F9	1.868(7)	1.896
Pt1–F5	1.957(7)	1.982	Pt2–F10	1.863(7)	1.899
Pt1–F6	1.895(9)	1.899			
Pt1–F7	1.905(11)	1.898			

Angle (°)	Experimental	Calculated	Angle (°)	Experimental	Calculated
F1–Xe1–F2	176.0(5)	177.4	F3–Pt2–F5^{ii}	89.9(4)	89.0
Xe1–F2–Pt1	123.0(4)	120.2	F3–Pt2–F8	177.4(4)	177.2
F2–Pt1–F3	90.5(4)	90.8	F3–Pt2–F9	92.2(4)	92.0
F2–Pt1–F4^{i}	90.8(3)	90.9	F3–Pt2–F10	90.7(4)	91.2
F2–Pt1–F5	177.2(4)	176.3	F4–Pt2–F5^{ii}	90.2(3)	90.4
F2–Pt1–F6	90.5(3)	90.1	F4–Pt2–F8	86.2(4)	85.9
F2–Pt1–F7	89.1(4)	89.5	F4–Pt2–F9	88.1(3)	87.1
F3–Pt1–F4^{i}	89.9(4)	89.4	F4–Pt2–F10	178.1(5)	176.8
F3–Pt1–F5	92.2(4)	92.9	F5^{ii}–Pt2–F8	90.2(4)	89.1
F3–Pt1–F6	90.4(4)	92.0	F5^{ii}–Pt2–F9	177.3(4)	177.3
F3–Pt1–F7	179.5(4)	178.7	F5^{ii}–Pt2–F10	90.0(3)	89.8
F4^{i}–Pt1–F5	90.1(3)	89.1	F8–Pt2–F9	87.6(4)	89.7
F4^{i}–Pt1–F6	178.7(3)	178.2	F8–Pt2–F10	91.9(3)	90.9
F4^{i}–Pt1–F7	90.3(4)	89.3	F9–Pt2–F10	91.6(3)	92.7
F5–Pt1–F6	88.6(3)	89.7	Pt1–F3–Pt2	133.0(4)	128.9
F5–Pt1–F7	88.3(4)	86.8	Pt1^{i}–F4–Pt2	132.8(5)	129.3
F6–Pt1–F7	89.4(4)	89.2	Pt1–F5–Pt2^{ii}	131.9(5)	128.9
F3–Pt2–F4	91.2(4)	92.0			

Symmetry codes: (i) $-x + 1$, $-y + 1$, $-z + 1$; (ii) $-x$, $-y + 1$, $-z + 1$

Table S3. Experimental (3D ED) and calculated (r^2SCAN-D3) geometrical parameters for $XeF_2{\cdot}2PdF_4$

Bond distance (Å)	Experimental	Calculated	Bond distance (Å)	Experimental	Calculated
Xe1–F1	1.892(5)	1.965	Pd2–F3	1.991(7)	1.997
Xe1–F2	2.320(4)	2.287	Pd2–F4	1.982(4)	1.998
Pd1–F2	1.894(4)	1.929	Pd2–F5^{ii}	2.020(4)	2.009
Pd1–F3	1.981(7)	1.991	Pd2–F8	1.851(8)	1.867
Pd1–F4^{i}	1.978(5)	1.980	Pd2–F9	1.866(4)	1.864
Pd1–F5	1.946(4)	1.953	Pd2–F10	1.844(4)	1.865
Pd1–F6	1.860(5)	1.868			
Pd1–F7	1.857(8)	1.867			

Angle (°)	Experimental	Calculated	Angle (°)	Experimental	Calculated
F1–Xe1–F2	178.0(3)	177.6	F3–Pd2–F5^{ii}	89.8(2)	89.0
Xe1–F2–Pd1	123.1(2)	120.6	F3–Pd2–F8	177.5(2)	176.7
F2–Pd1–F3	90.9(3)	91.1	F3–Pd2–F9	92.0(3)	92.1
F2–Pd1–F4^{i}	90.18(19)	90.6	F3–Pd2–F10	92.2(3)	92.1
F2–Pd1–F5	176.7(3)	176.6	F4–Pd2–F5^{ii}	89.14(16)	89.5
F2–Pd1–F6	89.3(2)	90.3	F4–Pd2–F8	87.1(3)	86.1
F2–Pd1–F7	89.8(3)	89.3	F4–Pd2–F9	88.58(17)	87.8
F3–Pd1–F4^{i}	89.5(2)	89.2	F4–Pd2–F10	176.9(4)	176.7
F3–Pd1–F5	92.3(2)	92.4	F5^{ii}–Pd2–F8	88.8(2)	89.0
F3–Pd1–F6	91.1(3)	92.2	F5^{ii}–Pd2–F9	177.2(3)	177.1
F3–Pd1–F7	179.0(2)	178.5	F5^{ii}–Pd2–F10	90.66(17)	90.8
F4^{i}–Pd1–F5	90.62(19)	89.5	F8–Pd2–F9	89.4(3)	89.8
F4^{i}–Pd1–F6	179.3(2)	178.3	F8–Pd2–F10	89.9(3)	90.6
F4^{i}–Pd1–F7	89.8(3)	89.4	F9–Pd2–F10	91.52(18)	91.9
F5–Pd1–F6	89.8(2)	89.5	Pd1–F3–Pd2	132.8(2)	129.5
F5–Pd1–F7	87.0(3)	87.3	Pd1^{i}–F4–Pd2	134.4(3)	131.1
F6–Pd1–F7	89.6(3)	89.2	Pd1–F5–Pd2^{ii}	132.0(3)	129.7
F3–Pd2–F4	90.9(2)	91.3			

Symmetry codes: (i) $-x + 1$, $-y + 1$, $-z + 1$; (ii) $-x$, $-y + 1$, $-z + 1$

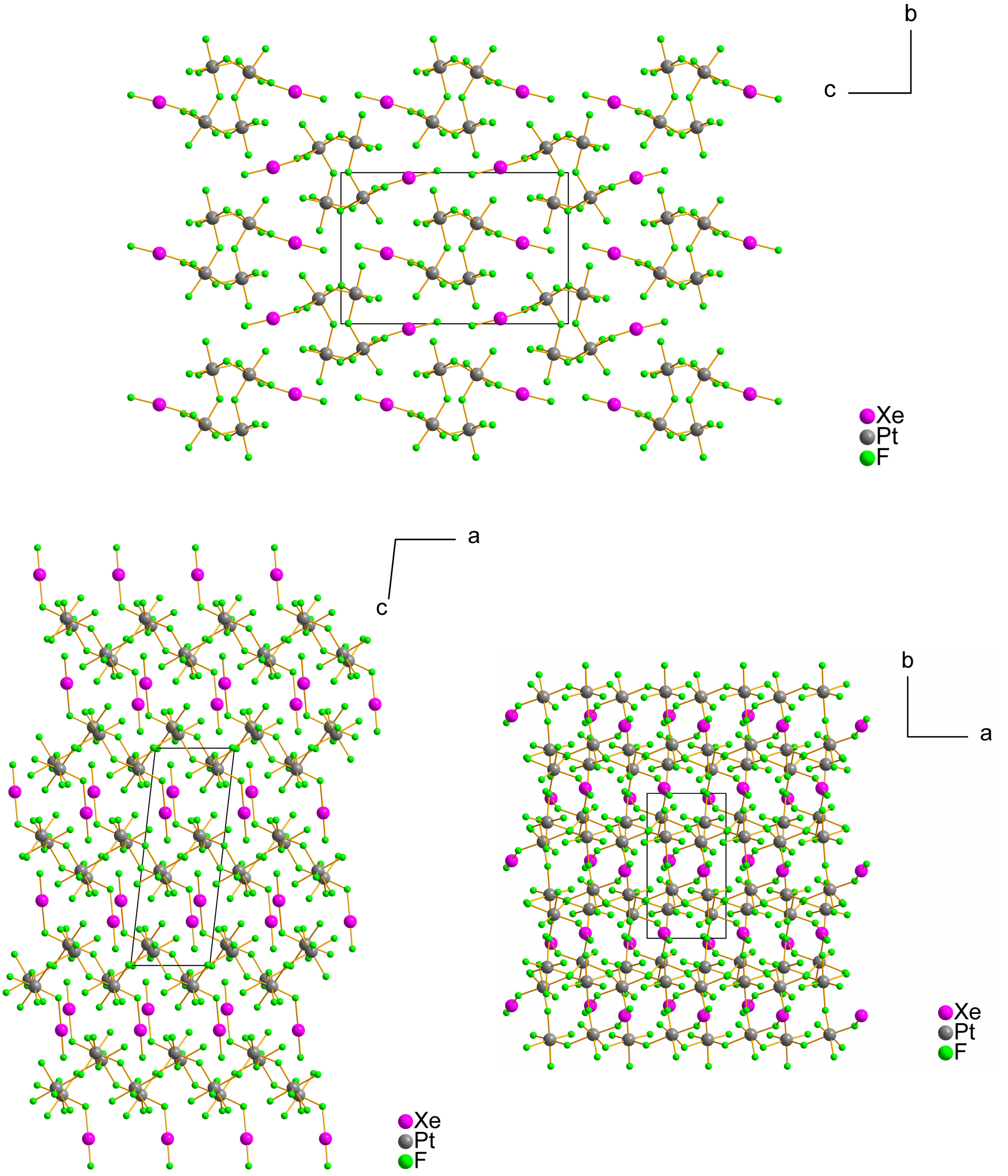

Figure S3. The crystal packing and the unit cell of $XeF_2{\cdot}2PtF_4$ viewed along the *a*- (top), *b*- (bottom left), and *c*-crystallographic axis (bottom right).

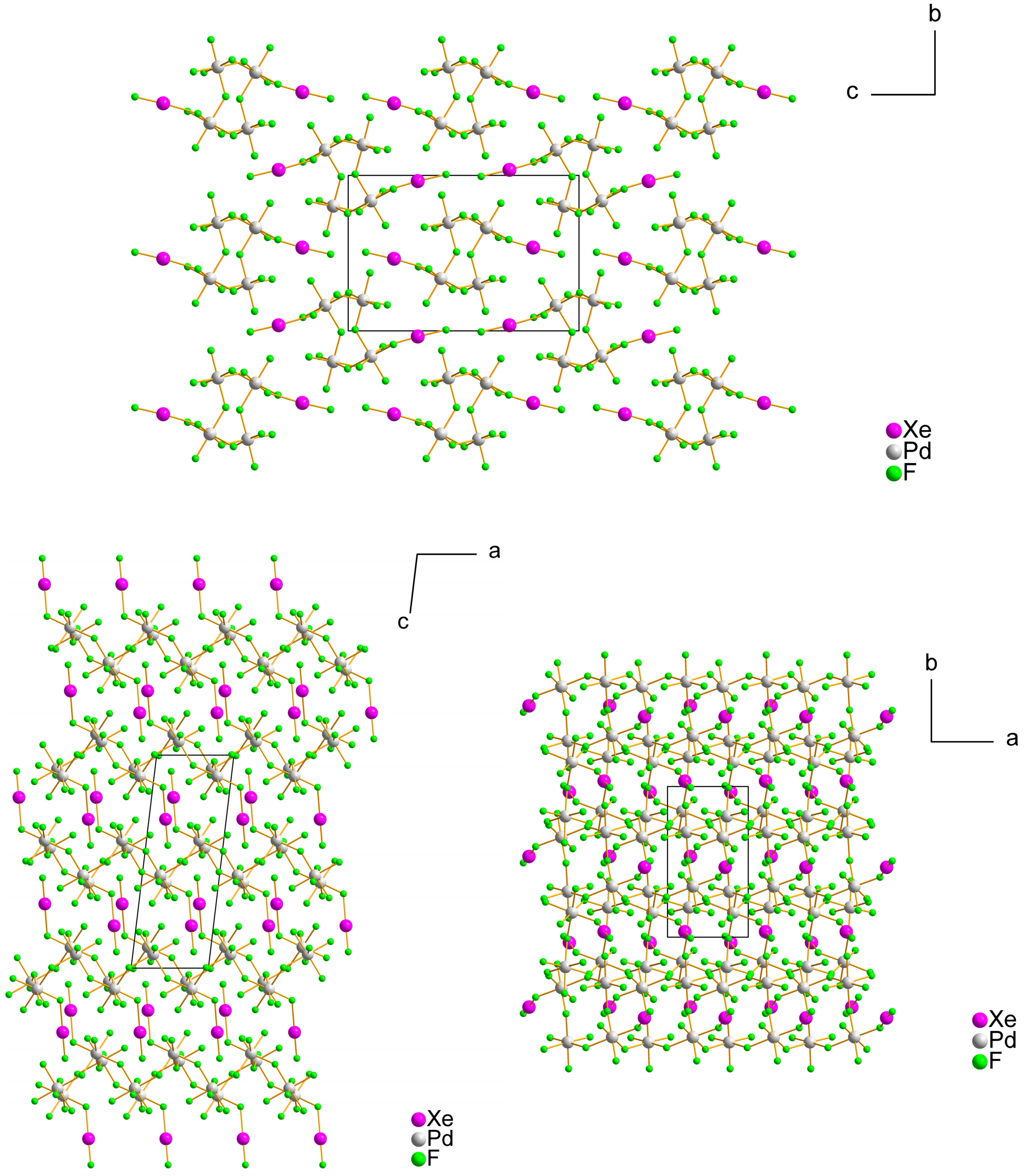

Figure S4. The crystal packing and the unit cell of $XeF_2 \cdot 2PdF_4$ viewed along the *a*- (top), *b*- (bottom left), and *c*-crystallographic axis (bottom right).

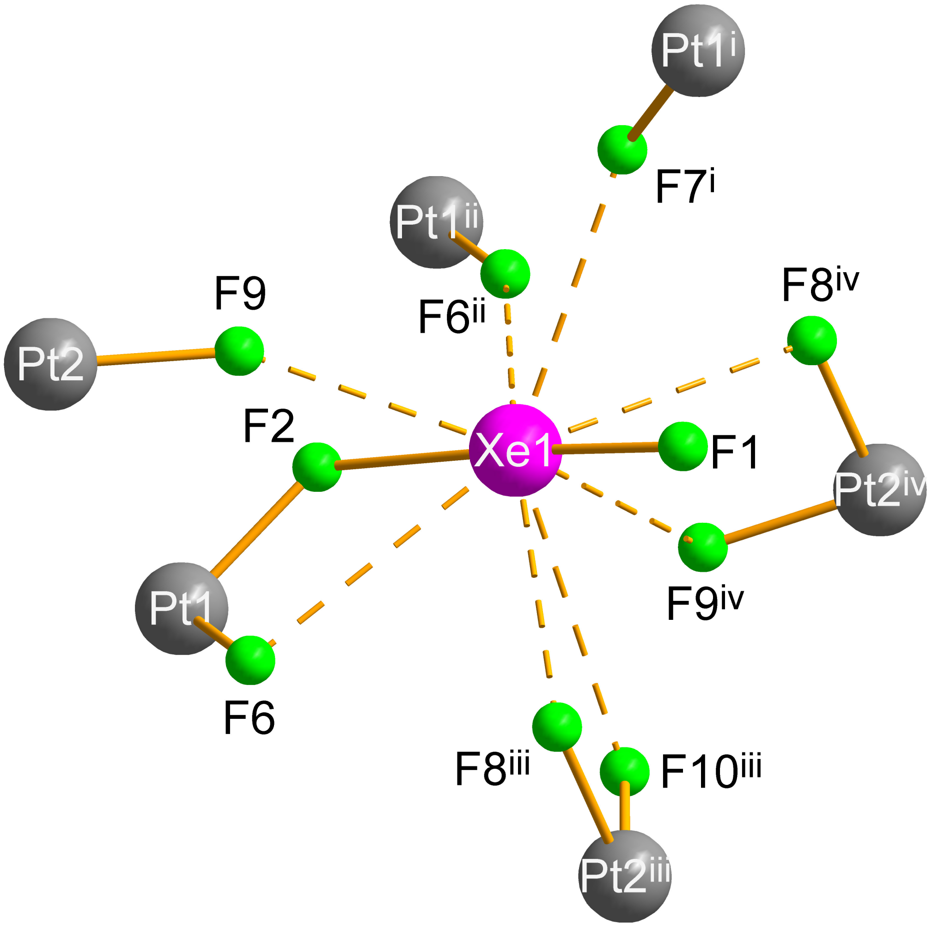

Figure S5. Xe1 coordination environment including contacts up to the sum of van der Waals radii for Xe and F (3.52 Å)[S1] in the crystal structure of $XeF_2{\cdot}2PtF_4$.

Table S4. Non-bonded Xe···F contacts shorter than the sum of van der Waals radii for Xe and F (3.52 Å)[S1] in the crystal structure of $XeF_2{\cdot}2PtF_4$.

Contact	Distance (Å)
Xe1···F7^{i}	3.019(10)
Xe1···F6^{ii}	3.070(8)
Xe1···F9	3.089(9)
Xe1···F8^{iii}	3.117(9)
Xe1···F6	3.267(7)
Xe1···F8^{iv}	3.348(8)
Xe1···F10^{iii}	3.399(12)
Xe1···F9^{iv}	3.421(10)

Symmetry codes:
(i) −*x* + 3/2, *y* + 1/2, −*z* + 3/2; (ii) *x* + 1, *y*, *z*; (iii) −*x* + 1/2, *y* − 1/2, −*z* + 3/2; (iv) −*x* + 3/2, *y* − 1/2, −*z* + 3/2.

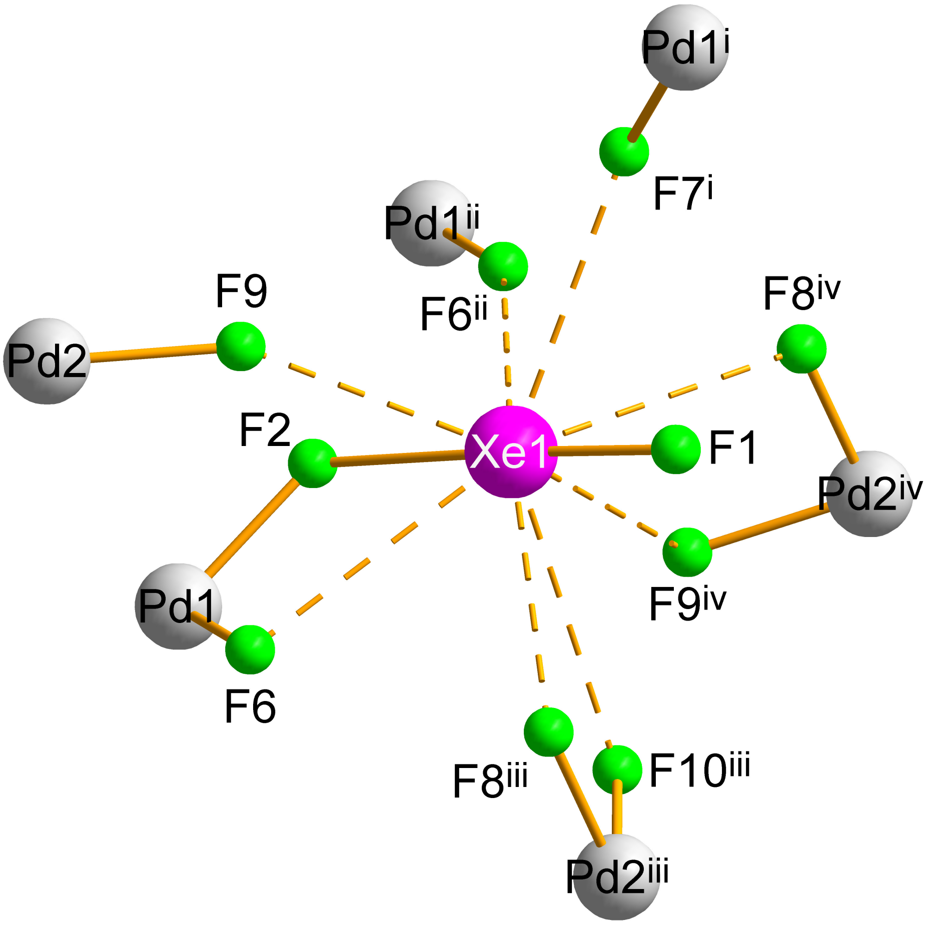

Figure S6. Xe1 coordination environment including contacts up to the sum of van der Waals radii of Xe and F (3.52 Å)[S1] in the crystal structure of $XeF_2 \cdot 2PdF_4$.

Table S5. Non-bonded Xe···F contacts shorter than the sum of van der Waals radii for Xe and F (3.52 Å)[S1] in the crystal structure of $XeF_2 \cdot 2PdF_4$.

Contact	Distance (Å)
Xe1···F9	3.049(6)
Xe1···$F8^{iv}$	3.083(5)
Xe1···$F7^{ii}$	3.092(7)
Xe1···$F6^{i}$	3.117(5)
Xe1···F6	3.191(4)
Xe1···$F8^{iii}$	3.355(5)
Xe1···$F10^{iv}$	3.359(9)
Xe1···$F9^{iii}$	3.427(7)

Symmetry codes:
(i) −*x* + 3/2, *y* + 1/2, −*z* + 3/2; (ii) *x* + 1, *y*, *z*; (iii) −*x* + 1/2, *y* − 1/2, −*z* + 3/2; (iv) −*x* + 3/2, *y* − 1/2, −*z* + 3/2.

Table S6. Experimental (SCXRD)[S2] and calculated (r^2SCAN-D3) unit-cell and geometrical parameters for $XeF_2{\cdot}CrF_4$

	Experimental	Calculated
Space group	$P2_1/n$	$P2_1/n$
a (Å)	7.666(2)	7.41160
b (Å)	7.268(5)	7.19939
c (Å)	9.901(3)	9.86022
β (°)	91.25(2)	90.0265
V (Å^3)	551.5	526.131
Z	4	4

Bond distance (Å)	Experimental	Calculated	Bond distance (Å)	Experimental	Calculated
Xe1–F1	2.13(2)	2.159	Cr1–F3^i	1.91(2)	1.910
Xe1–F2	1.93(2)	1.993	Cr1–F4	1.75(2)	1.772
Cr1–F1	2.00(2)	2.003	Cr1–F5	1.73(2)	1.761
Cr1–F3	1.88(2)	1.905	Cr1–F6	1.71(2)	1.727

Angle (°)	Experimental	Calculated	Angle (°)	Experimental	Calculated
F1–Xe1–F2	176.2(1)	176.2	F3–Cr1–F6	90.2(7)	90.9
F1–Cr1–F3	85.9(6)	85.8	F3^i–Cr1–F4	88.4(7)	87.2
F1–Cr1–F3^i	89.8(6)	88.7	F3^i–Cr1–F5	90.6(7)	91.8
F1–Cr1–F4	82.5(7)	82.8	F3^i–Cr1–F6	94.0(7)	94.6
F1–Cr1–F5	79.8(7)	80.8	F4–Cr1–F5	162.3(3)	163.5
F1–Cr1–F6	179.9(1)	178.7	F4–Cr1–F6	97.6(8)	98.5
F3–Cr1–F3^i	175.6(1)	174.2	F5–Cr1–F6	100.1(8)	98.0
F3–Cr1–F4	89.8(7)	90.3	Cr1–F3–Cr1^{ii}	147.3(8)	141.8
F3–Cr1–F5	89.9(7)	89.1			

Symmetry codes: (i) −*x* + 3/2, *y* − 1/2, −*z* + 3/2; (ii) −*x* + 3/2, *y* − 1/2, −*z* + 3/2

Table S7. Experimental (SCXRD)[S3] and calculated (r^2SCAN-D3) geometrical parameters for $XeF_2{\cdot}MnF_4$

	Experimental	Calculated
Space group	$P2_1/n$	$P2_1/n$
a (Å)	9.6430(5)	9.63134
b (Å)	10.9859(4)	10.9299
c (Å)	9.7927(3)	9.67481
β (°)	96.979(4)	96.5082
V (Å^3)	1029.72(7)	1011.90
Z	4	4

Bond distance (Å)	Experimental	Calculated	Bond distance (Å)	Experimental	Calculated
Xe1–F1	1.906(3)	1.986	Xe2–F8	1.910(3)	1.988
Xe1–F2	2.176(3)	2.200	Xe2–F9	2.180(3)	2.206
Mn1–F2	1.902(3)	1.911	Mn2–F3	1.900(3)	1.905
Mn1–F3	1.889(3)	1.900	Mn2–F4^{i}	1.903(3)	1.907
Mn1–F4	1.887(3)	1.894	Mn2–F9	1.928(3)	1.943
Mn1–F5	1.741(3)	1.744	Mn2–F10	1.729(3)	1.739
Mn1–F6	1.722(3)	1.737	Mn2–F11	1.725(3)	1.742
Mn1–F7	1.721(3)	1.740	Mn2–F12	1.720(3)	1.734

Angle (°)	Experimental	Calculated	Angle (°)	Experimental	Calculated
F1–Xe1–F2	178.52(14)	177.5	F3–Mn2–F9	84.25(13)	84.3
F8–Xe2–F9	177.48(14)	177.0	F4^{i}–Mn2–F9	82.96(13)	83.3
F6–Mn1–F3	174.66(16)	174.6	F11–Mn2–F3	91.03(14)	91.0
F6–Mn1–F4	89.27(15)	88.9	F11–Mn2–F4^{i}	92.27(14)	92.1
F6–Mn1–F2	90.44(17)	90.9	F11–Mn2–F10	93.72(16)	94.0
F6–Mn1–F5	92.53(16)	92.7	F11–Mn2–F9	173.54(14)	173.7
F3–Mn1–F2	86.35(13)	86.1	F10–Mn2–F3	90.43(15)	90.7
F4–Mn1–F3	86.24(13)	86.4	F10–Mn2–F4^{i}	172.62(15)	172.7
F4–Mn1–F2	85.85(13)	85.1	F10–Mn2–F9	90.73(16)	90.4
F5–Mn1–F3	91.64(14)	91.8	F12–Mn2–F3	172.69(14)	172.1
F5–Mn1–F4	173.82(14)	173.4	F12–Mn2–F4^{i}	89.76(14)	89.4
F5–Mn1–F2	88.23(14)	88.5	F12–Mn2–F11	94.42(15)	94.8
F7–Mn1–F6	93.92(18)	94.1	F12–Mn2–F10	94.08(16)	94.2
F7–Mn1–F3	89.15(14)	88.6	F12–Mn2–F9	89.93(14)	89.5
F7–Mn1–F4	92.28(14)	92.2	Mn1–F3–Mn2	149.24(17)	145.8
F7–Mn1–F2	175.23(15)	174.2	Mn1–F4–Mn2^{i}	147.93(17)	145.5
F7–Mn1–F5	93.49(15)	94.0	Mn1–F2–Xe1	127.80(15)	124.8
F3–Mn2–F4^{i}	85.14(13)	85.0	Mn2–F9–Xe2	121.48(15)	119.6

Symmetry code: (i) $-x + 1, -y + 1, -z + 1$.

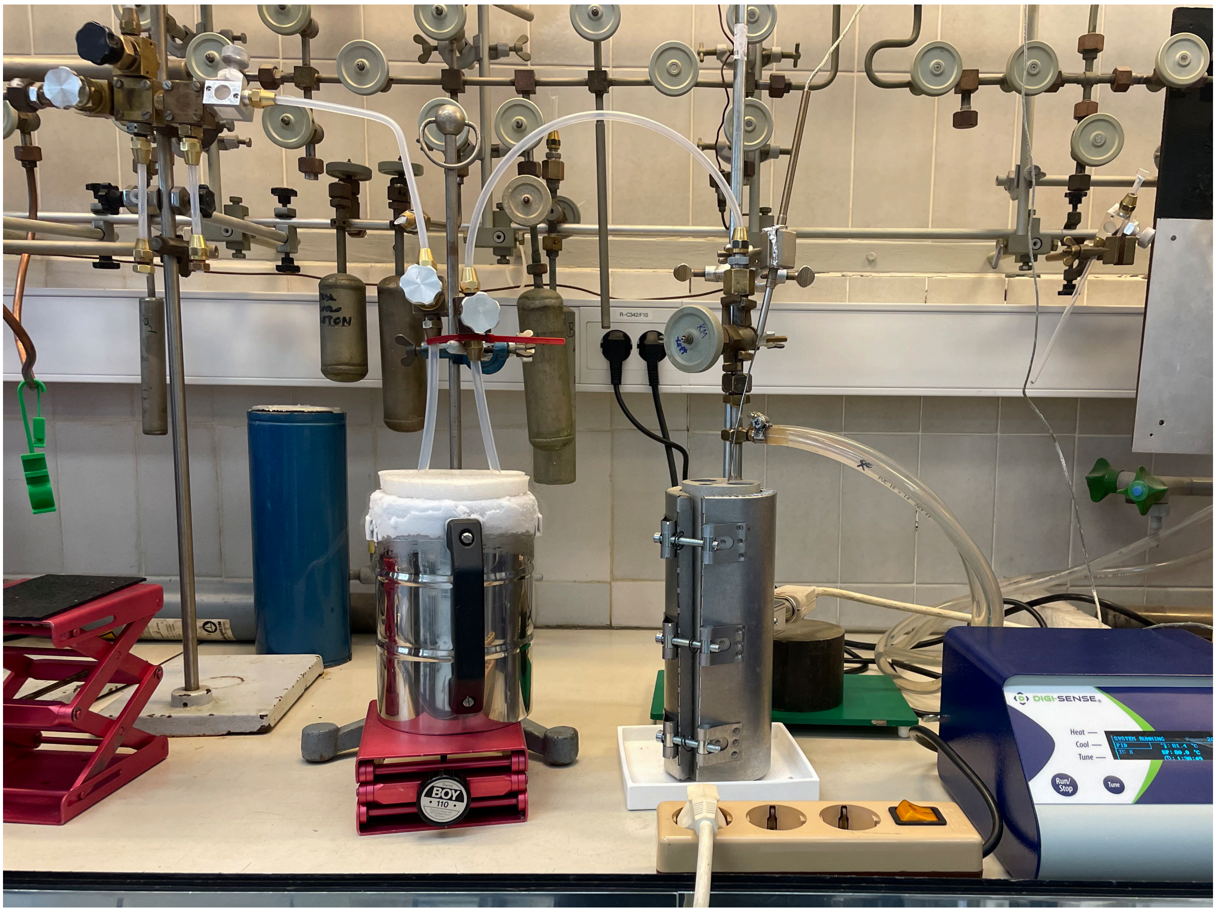

Figure S7. Experimental setup used for the pyrolysis of the XeF_2–PtF_4 and XeF_2–PdF_4 adducts. The nickel reactor was connected to a vacuum line via FEP connectors (i.d. 3 mm; o.d. 6 mm) and a U-shaped FEP trap (i.d. 6 mm; o.d. 8 mm), which were previously passivated by F_2. The trap was immersed in liquid nitrogen, allowing for deposition of the released XeF_2. The reactor was placed in a furnace, controlled by a temperature controller (Digi-Sense TC9100). The upper part of the reactor was cooled by water circulated through a cooling ring, with the temperature of the circulating water kept constant at 20 °C by the use of a cooling bath thermostat (Huber KISS K6).

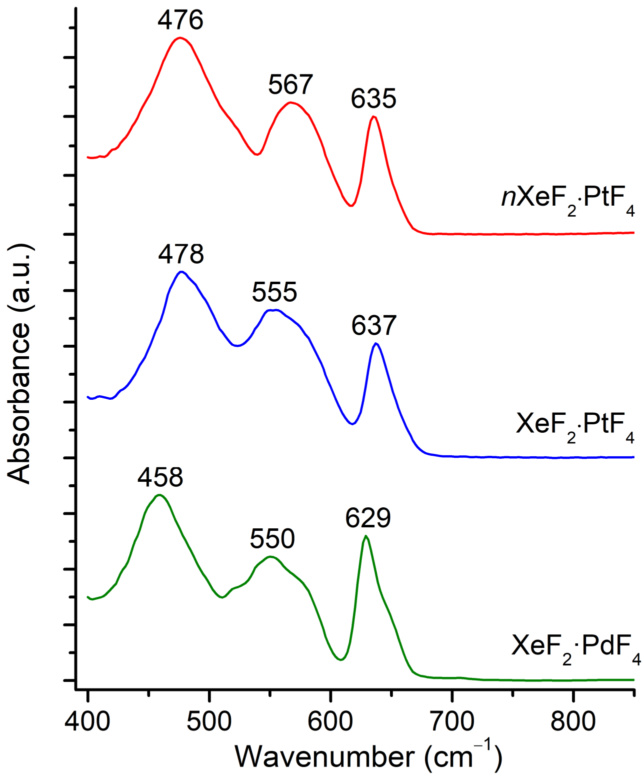

Figure S8. ATR-IR spectra recorded on powdered samples of the product of Pt oxidation by XeF_2 in aHF, $n$$XeF_2$·$PtF_4$ (n > 1) (top), the product of its pyrolysis under dynamic vacuum at 100 °C, XeF_2·PtF_4 (middle), and XeF_2·PdF_4 (bottom).

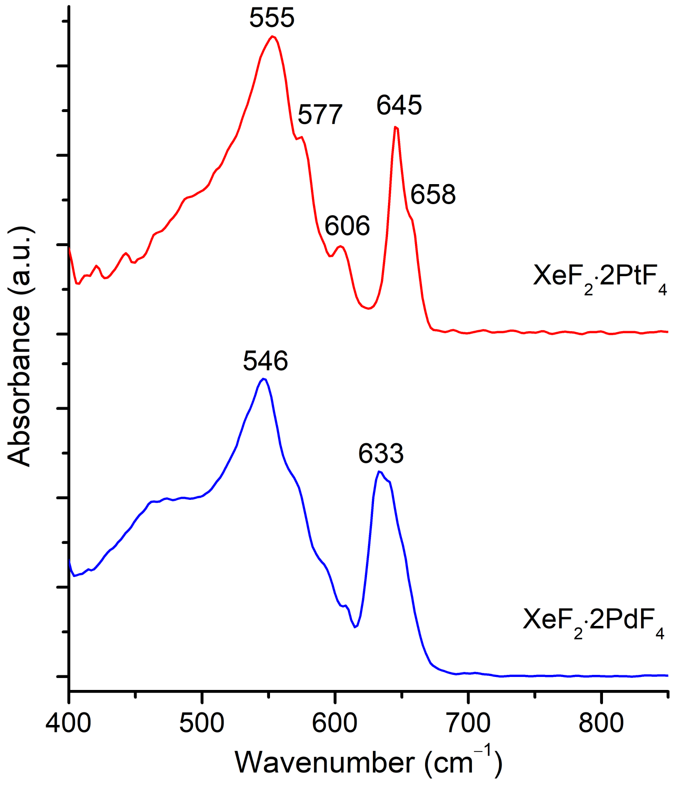

Figure S9. ATR-IR spectra recorded on powdered samples of XeF_2·2PtF_4 (top) and XeF_2·2PdF_4 (bottom).

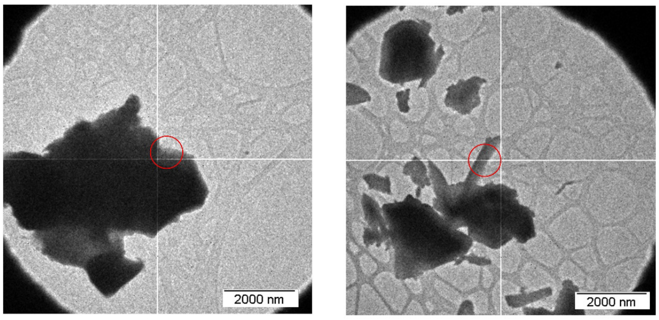

(a) (b)

Figure S10. TEM images of $XeF_2{\cdot}2PtF_4$ crystallites that were used for the 3D ED experiment. The red circle represents the beam size and the location used for the 3D ED data collection.

Table S8. 3D ED data collection and results for $XeF_2{\cdot}2PtF_4$

**Collection information**			
3D ED collection method	Continuous-rotation data collection from two crystals		
Tilt information [a]	Crystal		α_{min}, α_{max}, $\Delta\alpha$ (°)
	a		−59.59, 39.24, 0.30
	b		−27.09, 59.71, 0.30
Exposure time (ms)	405, 405		
Beam diameter (nm)	870, 870		
Camera length (mm)	1500, 1200		
Electron dose information	Crystal	Flux density (e^- $Å^{-2}s^{-1}$) [b]	Fluence (e^- $Å^{-2}$) [c]
	a	0.0173	2.31
	b	0.0173	2.03
**Crystal information**			
Empirical formula	$XePt_2F_{10}$		
Formula unit, *Z*	4		
Space group	$P2_1/n$		
a, *b*, *c* (Å)	5.4189(3), 9.8998(8), 14.9993(9)		
α, β, γ (°)	90, 96.506(5), 90		
V ($Å^3$)	799.5(9)		
Apparent mosaicities (°)	0.1257, 0.1735		
Completeness (%)	97.4		
**Dynamical refinement statistics** [d]			
$\sin(\theta_{max})/\lambda$ ($Å^{-1}$)	0.70		
N_{obs}, N_{all}	3786, 8675		
Parameters	224		
R_{obs}, wR_{obs}	0.0952, 0.1016		
R_{all}, wR_{all}	0.1362, 0.1120		
$\Delta V(r)_{min}$, $\Delta V(r)_{max}$ (e/Å)	−0.27, 0.29		

[a] Reported values are after the frame orientation optimization in *PETS2*.[S4]

[b] Defined as particles (electrons) delivered per unit area per unit time.[S5]

[c] Defined as particles (electrons) delivered per unit area.[S5]

[d] Kinematical refinement statistics were omitted because kinematical refinements were done only once and were not optimized before performing dynamical refinements.

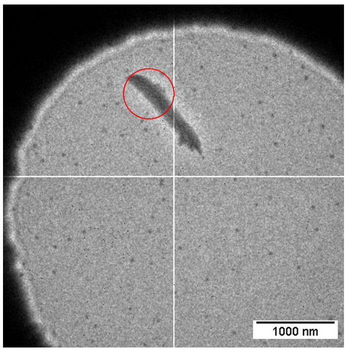

Figure S11. TEM image of $XeF_2 \cdot 2PdF_4$ crystallite used for 3D ED experiment. The red circle represents the beam size and location used for the 3D ED data collection.

Table S9. 3D ED data collection and results for $XeF_2 \cdot 2PdF_4$

Collection information		
3D ED collection method	Continuous-rotation data collection from one crystal	
Tilt information [a]	α_{min}, α_{max}, $\Delta\alpha$ (°)	
	−55.16, 44.78, 0.30	
Exposure time (ms)	504	
Beam diameter (nm)	650	
Camera length (mm)	1500	
Electron dose information	Flux density (e^{-} $Å^{-2}s^{-1}$) [b]	Fluence (e^{-} $Å^{-2}$) [c]
	0.0132	2.22
**Crystal information**		
Empirical formula	$XePd_2F_{10}$	
Formula unit, *Z*	4	
Space group	$P2_1/n$	
a, *b*, *c* (Å)	5.3722(5), 9.959(3), 14.9091(8)	
α, β, γ (°)	90, 96.842(6), 90	
V ($Å^3$)	792.0(2)	
Apparent mosaicity (°)	0.1255	
Completeness (%)	72.0%	
**Dynamical refinement statistics** [d]		
$\sin(\theta_{max})/\lambda$ ($Å^{-1}$)	0.70	
N_{obs}, N_{all}	2548, 4356	
Parameters	170	
R_{obs}, wR_{obs}	0.0657, 0.0630	
R_{all}, wR_{all}	0.0951, 0.0685	
$\Delta V(r)_{min}$, $\Delta V(r)_{max}$ (e/Å)	−0.09, 0.10	

[a] Reported values are after the frame orientation optimization in *PETS2*.[S4]

[b] Defined as particles (electrons) delivered per unit area per unit time.[S5]

[c] Defined as particles (electrons) delivered per unit area.[S5]

[d] Kinematical refinement statistics were omitted because kinematical refinements were done only once and were not optimized before performing dynamical refinements.

Table S10. General microscope information

Microscope	FEI Tecnai G^2 20 transmission electron microscope
Radiation source	LaB_6
Detector type	Medipix 3 hybrid pixel detector ASI CheeTah<br>(512 × 512 pixels, 24-bit dynamic range)
Accelerating voltage (kV)	200
Wavelength, λ (Å)	0.02508
Probe type	Microdiffraction
Detector pixel size (µm)	55 × 55
Calibration constant (Å^{-1}/pixel)	0.005664
Measurement temperature, *T* (K)	100

Table S11. Coordinates of the DFT-calculated (r^{2}SCAN-D3) crystal structures

$XeF_2{\cdot}2PtF_4$

($P2_1/n$, a = 5.41786 Å, b = 9.70193 Å, c = 14.95579 Å, β = 96.3704°, V = 781.28 Å^3)

Atom	*x*	*y*	*z*
**Xe1**	0.71265	0.03436	0.29763
**Pt1**	0.18556	0.66549	0.40118
**Pt2**	0.73226	0.70496	0.56224
**F1**	0.78262	0.98933	0.42630
**F2**	0.86007	0.59682	0.35224
**F3**	0.75401	0.50586	0.52252
**F4**	0.03758	0.75750	0.50317
**F5**	0.51234	0.74594	0.44596
**F6**	0.32969	0.58388	0.30390
**F7**	0.12733	0.82845	0.33184
**F8**	0.71584	0.89399	0.59379
**F9**	0.94860	0.67221	0.66864
**F10**	0.44512	0.66604	0.61994

$XeF_2{\cdot}2PdF_4$

($P2_1/n$, a = 5.34201 Å, b = 9.70082 Å, c = 14.80389 Å, β = 96.6662°, V = 761.98 Å^3)

Atom	*x*	*y*	*z*
**Xe1**	0.78462	0.53539	0.20139
**Pd1**	0.18461	0.66486	0.40145
**Pd2**	0.26753	0.29890	0.43573
**F1**	0.71550	0.48896	0.07166
**F2**	0.85764	0.59826	0.35105

F3	0.24562	0.49462	0.47582
F4	0.03494	0.75174	0.50366
F5	0.51181	0.74224	0.44806
F6	0.32963	0.58798	0.30419
F7	0.12565	0.82712	0.33448
F8	0.28216	0.11276	0.40463
F9	0.05019	0.33142	0.33001
F10	0.55018	0.33570	0.37664

$XeF_2 \cdot CrF_4$ (tetrameric ring)

($P2_1/n$, a = 9.57279 Å, b = 11.07794 Å, c = 9.75114 Å, β = 97.3704°, V = 1025.53 Å^3)

Atom	*x*	*y*	*z*
Xe1	0.54091	0.78080	−0.04736
Xe2	0.43119	0.30498	0.53765
Cr1	0.28829	0.01914	0.64314
Cr2	0.67575	0.00792	0.72916
F1	0.49208	0.03452	0.63831
F2	0.29721	0.86092	0.62318
F3	0.48063	0.71635	0.12647
F4	0.55963	0.39734	0.43009
F5	0.29039	0.20046	0.64997
F6	0.10579	0.02187	0.63158
F7	0.72475	0.15510	0.69119
F8	0.32846	0.02108	0.82242
F9	0.71348	−0.04961	0.54650
F10	0.63393	0.04784	0.89462
F11	0.59862	0.84796	0.75613
F12	0.84096	−0.05026	0.78894

$XeF_2 \cdot CrF_4$ (*cis*-chains)

($P2_1/n$, a = 9.78454 Å, b = 4.89241 Å, c = 11.12479 Å, β = 92.2555°, V = 532.13 Å^3)

Atom	*x*	*y*	*z*
Xe1	0.74058	−0.00466	0.05808
Cr1	0.61802	0.54960	0.79218
F1	0.52056	0.35844	0.88927
F2	0.75973	0.31935	0.16728
F3	0.52194	0.84994	0.80173
F4	0.76029	0.72607	0.71385

F5	0.53983	0.44893	0.65469
F6	0.72900	0.64478	−0.06135

XeF_2·CrF_4 (*trans*-chains)

($P2_1/n$, a = 7.41160 Å, b = 7.19939 Å, c = 9.86022 Å, β = 90.0265°, V = 526.13 Å^3)

Atom	*x*	*y*	*z*
Xe1	0.76888	0.34083	0.61442
Cr1	0.74730	0.41348	0.24080
F1	0.88098	0.35901	0.41284
F2	0.67895	0.33438	0.80486
F3	0.72279	0.65998	0.30988
F4	0.55741	0.35377	0.34078
F5	−0.03127	0.45801	0.18499
F6	0.63658	0.46013	0.09045

XeF_2·MnF_4 (tetrameric ring)

($P2_1/n$, a = 9.63134 Å, b = 10.92988 Å, c = 9.67481 Å, β = 96.5082°, V = 1011.90 Å^3)

Atom	*x*	*y*	*z*
Xe1	0.04587	0.22484	0.45841
Xe2	0.46322	0.69144	0.56448
Mn1	0.35851	−0.02242	0.70231
Mn2	0.27057	−0.00609	0.32710
F1	0.36131	−0.04264	0.50707
F2	0.38386	0.13472	0.68805
F3	0.87122	0.29000	0.52154
F4	0.57510	0.60108	0.43725
F5	0.34553	0.80017	0.70403
F6	0.37017	−0.02198	0.88250
F7	0.30876	0.84615	0.27599
F8	0.17728	−0.01120	0.68020
F9	0.44580	0.05384	0.28609
F10	0.10793	−0.04623	0.37637
F11	0.24419	0.15462	0.39752
F12	0.19894	0.03828	0.16187

$XeF_2 \cdot MnF_4$ (*cis*-chains)

($P2_1/n$, a = 9.66091 Å, b = 4.93096 Å, c = 11.13972 Å, β = 92.4872°, V = 530.17 Å^3)

Atom	*x*	*y*	*z*
Xe1	0.74191	−0.01015	0.05876
Mn1	0.62464	0.55532	0.79825
F1	0.51395	0.37160	0.88633
F2	0.75827	0.31110	0.16697
F3	0.53208	0.85371	0.81525
F4	0.75798	0.74582	0.70997
F5	0.54113	0.46483	0.66227
F6	0.73688	0.63555	−0.05916

$XeF_2 \cdot MnF_4$ (*trans*-chains)

($P2_1/n$, a = 7.41324 Å, b = 7.06513 Å, c = 9.77662 Å, β = 91.7227°, V = 511.82 Å^3)

Atom	*x*	*y*	*z*
Xe1	0.76574	0.33141	0.61325
Mn1	0.74846	0.41692	0.24313
F1	0.87969	0.36255	0.40977
F2	0.68015	0.31804	0.80335
F3	0.72655	0.66418	0.31055
F4	0.54841	0.36339	0.33055
F5	−0.04050	0.46708	0.17305
F6	0.63341	0.46572	0.09054

$XeF_2 \cdot PtF_4$ (tetrameric ring)

($P2_1/n$, a = 9.62961 Å, b = 11.15053 Å, c = 9.87216 Å, β = 93.7564°, V = 1057.75 Å^3)

Atom	*x*	*y*	*z*
Xe1	0.54265	0.76925	−0.03043
Xe2	0.41447	0.31996	0.53319
Pt1	0.29669	0.03087	0.63633
Pt2	0.67010	−0.01292	0.73327
F1	0.50424	0.05710	0.62342
F2	0.31136	0.86330	0.60150
F3	0.49597	0.72072	0.15494
F4	0.53355	0.40433	0.40748
F5	0.28230	0.20961	0.66710
F6	0.10113	0.01460	0.64351

F7	0.76045	0.13697	0.71088
F8	0.32579	−0.00213	0.82503
F9	0.74457	−0.07609	0.56075
F10	0.60913	0.04024	−0.09799
F11	0.57527	0.82924	0.75737
F12	0.82839	−0.07809	0.83166

$XeF_2 \cdot PtF_4$ (*cis*-chains)

(*P*2$_1$/*n*, *a* = 9.79474 Å, *b* = 5.03849 Å, *c* = 11.45661 Å, β = 94.9080°, *V* = 563.32 Å^3)

Atom	*x*	*y*	*z*
Xe1	0.73671	0.00236	0.06538
Pt1	0.62756	0.54632	0.79895
F1	0.50141	0.37370	0.89060
F2	0.75689	0.31724	0.17013
F3	0.52101	0.86099	0.80007
F4	0.75954	0.71052	0.69374
F5	0.53485	0.42949	0.65526
F6	0.73403	0.64497	−0.04956

$XeF_2 \cdot PtF_4$ (*trans*-chains)

(*P*2$_1$/*n*, *a* = 7.42548 Å, *b* = 7.21452 Å, *c* = 10.03173 Å, β = 92.7759°, *V* = 536.78 Å^3)

Atom	*x*	*y*	*z*
Xe1	0.76393	0.32162	0.61607
Pt1	0.75298	0.42125	0.25000
F1	0.89265	0.36150	0.42049
F2	0.66993	0.30843	0.79756
F3	0.72859	0.67070	0.32872
F4	0.53193	0.36828	0.33819
F5	−0.02489	0.47635	0.16517
F6	0.62691	0.48118	0.08664

$XeF_2 \cdot PdF_4$ (tetrameric ring)

(*P*2$_1$/*n*, *a* = 9.60943 Å, *b* = 11.08416 Å, *c* = 9.78347 Å, β = 94.2073°, *V* = 1039.25 Å^3)

Atom	*x*	*y*	*z*
Xe1	0.04049	0.27100	0.46660
Xe2	0.41845	0.68245	0.53462
Pd1	0.29789	−0.02983	0.63750

**Pd2**	0.32954	−0.01072	0.26779
**F1**	0.50274	0.05577	0.62532
**F2**	0.31095	0.8644	0.60203
**F3**	0.49153	0.71807	0.15076
**F4**	0.53738	0.40332	0.40893
**F5**	0.28625	0.20853	0.66778
**F6**	0.10495	0.0158	0.64307
**F7**	0.75849	0.13773	0.70973
**F8**	0.3241	−0.00081	0.82552
**F9**	0.73902	−0.07431	0.55936
**F10**	0.61223	0.04123	−0.09959
**F11**	0.57675	0.83184	0.75614
**F12**	0.82995	−0.07389	0.8264

$XeF_2{\cdot}PdF_4$ (*cis*-chains)

($P2_1/n$, a = 9.76696 Å, b = 5.00018 Å, c = 11.35426 Å, β = 94.4471°, V = 552.83 Å^3)

Atom	*x*	*y*	*z*
**Xe1**	0.73764	0.00034	0.06377
**Pd1**	0.62723	0.54877	0.79830
**F1**	0.50216	0.37220	0.88759
**F2**	0.75756	0.31697	0.16940
**F3**	0.52220	0.85987	0.80260
**F4**	0.75946	0.71702	0.69613
**F5**	0.53443	0.43877	0.65617
**F6**	0.73457	0.64454	−0.05115

$XeF_2{\cdot}PdF_4$ (*trans*-chains)

($P2_1/n$, a = 7.40384 Å, b = 7.18702 Å, c = 9.92387 Å, β = 92.7586°, V = 527.45 Å^3)

Atom	*x*	*y*	*z*
**Xe1**	0.76493	0.32381	0.61510
**Pd1**	0.75207	0.42187	0.24849
**F1**	0.89136	0.36188	0.41871
**F2**	0.67403	0.30843	0.79923
**F3**	0.73049	0.67084	0.32550
**F4**	0.53402	0.36962	0.33602
**F5**	−0.02814	0.47543	0.16524
**F6**	0.62620	0.48014	0.08667

References

(S1) Alvarez, S. A Cartography of the van Der Waals Territories. *Dalton Trans.* **2013**, *42* (24), 8617–8636. https://doi.org/10.1039/C3DT50599E

(S2) Lutar, K.; Leban, I.; Ogrin, T.; Žemva, B. $XeF_2 \cdot CrF_4$ and $(XeF_5^+CrF_5^-)_4 \cdot XeF_4$: Syntheses, Crystal Structures and Some Properties. *Eur. J. Solid State Inorg. Chem.* **1992**, *29*, 713–727.

(S3) Motaln, K.; Gurung, K.; Brázda, P.; Kokalj, A.; Radan, K.; Dragomir, M.; Žemva, B.; Palatinus, L.; Lozinšek, M. Reactive Noble-Gas Compounds Explored by 3D Electron Diffraction: XeF_2–MnF_4 Adducts and a Facile Sample Handling Procedure. *ACS Cent. Sci.* **2024**, *10* (9), 1733–1741. https://doi.org/10.1021/acscentsci.4c00815

(S4) Palatinus, L.; Brázda, P.; Jelínek, M.; Hrdá, J.; Steciuk, G.; Klementová, M. Specifics of the Data Processing of Precession Electron Diffraction Tomography Data and Their Implementation in the Program *PETS2.0*. *Acta Crystallogr. B* **2019**, *75* (4), 512–522. https://doi.org/10.1107/S2052520619007534

(S5) Saha, A.; Nia, S. S.; Rodríguez, J. A. Electron Diffraction of 3D Molecular Crystals. *Chem. Rev.* **2022**, *122* (17), 13883–13914. https://doi.org/10.1021/acs.chemrev.1c00879
